# Predictive Models of Type 1 Diabetes Progression: Understanding T-Cell Cycles and Their Implications on Autoantibody Release

**DOI:** 10.1371/journal.pone.0093326

**Published:** 2014-04-04

**Authors:** Majid Jaberi-Douraki, Massimo Pietropaolo, Anmar Khadra

**Affiliations:** 1 Department of Physiology, McGill University, Montreal, QC, Canada; 2 Laboratory of Immunogenetics, University of Michigan, Ann Arbor, Michigan, United States of America; La Jolla Institute for Allergy and Immunology, United States of America

## Abstract

Defining the role of T-cell avidity and killing efficacy in forming immunological response(s), leading to relapse-remission and autoantibody release in autoimmune type 1 diabetes (T1D), remains incompletely understood. Using competition-based population models of T- and B-cells, we provide a predictive tool to determine how these two parametric quantities, namely, avidity and killing efficacy, affect disease outcomes. We show that, in the presence of T-cell competition, successive waves along with cyclic fluctuations in the number of T-cells are exhibited by the model, with the former induced by transient bistability and the latter by transient periodic orbits. We hypothesize that these two immunological processes are responsible for making T1D a relapsing-remitting disease within prolonged but limited durations. The period and the number of peaks of these two processes differ, making them potential candidates to determine how plausible waves and cyclic fluctuations are in producing such effects. By assuming that T-cell and B-cell avidities are correlated, we demonstrate that autoantibodies associated with the higher avidity T-cell clones are first to be detected, and they reach their detectability level faster than those associated with the low avidity clones, independent of what T-cell killing efficacies are. Such outcomes are consistent with experimental observations in humans and they provide a rationale for observing rapid and slow progressors of T1D in high risk subjects. Our analysis of the models also reveals that it is possible to improve disease outcomes by unexpectedly increasing the avidity of certain subclones of T-cells. The decline in the number of 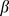-cells in these cases still occurs, but it terminates early, leaving sufficient number of functioning 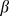-cells in operation and the affected individual asymptomatic. These results indicate that the models presented here are of clinical relevance because of their potential use in developing predictive algorithms of rapid and slow progression to clinical T1D.

## Introduction

Type 1 diabetes (T1D), the immune mediated form of diabetes, is a relatively common disorder that results from the destruction of insulin-producing 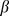-cells of the pancreas [1]–[12]. It is widely acknowledged that the demolition of 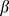-cells in genetically susceptible individuals is caused by the activation of cytotoxic T lymphocytes (CTLs) and helper T-cells (including CD8^+^ and CD4^+^ T-cells) whose T-cell receptors (TCRs) are reactive to 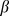-cell-specific autoantigens expressed as peptide-major histocompatibility complexes (pMHCs) on antigen presenting cells (APCs). The binding kinetics of TCRs with pMHCs has been extensively studied [13]. The progression of T1D is, in general, associated with the presence of autoreactive T-cells specific for 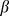-cell autoantigens, and a sequence of pancreatic anti-islet autoantibodies which can be marked by their presence for years prior to the inception of abnormal hyperglycemia (an excess of glucose in the bloodstream). It was previously thought that T-cells are solely implicated in T1D onset and progression, but new evidence from studies of nonobese diabetic (NOD) mouse model suggests that antibody-secreting mature B-lymphocytes (or plasma-cells) also contribute to pathogenesis [14].

The direct visualization of CD4^+^ T-cells by flow cytometry can now be achieved using MHC class II tetramers [15]. In prediabetic patients, CD4^+^ T-cell responses directed against proinsulin and glutamic acid decarboxylase 555–567 (GAD 555) have been reported [16], [17]. Moreover, Standifer et al. [18] observed that a cohort of autoantibody-positive, at-risk subjects exhibited a significantly increased frequency of CD8^+^ T-cells responding to an epitope of prepro-islet amyloid polypeptide. In fact, it was confirmed that CD8^+^ T-cells reactive to multiple HLA-A2-restricted 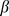-cell epitopes, including insulin B(10–18), islet antigen IA-2(797–805) and islet-specific glucose-6-phosphatase catalytic subunit-related protein IGRP(265–273), can be simultaneously detected with high frequency in recent-onset diabetic patients but rarely in healthy control subjects [19]. Islet-specific autoantigens play a crucial role in directing the progression of 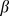-cell-specific autoimmune responses. CTLs as effectors kill 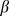-cells that are erroneously marked as contaminated with viral particles during adaptive immune response. Helper T-cells, on the other hand, secrete cytokines that help other cells of the immune system become fully activated effector cells. In T1D, some subsets of helper T-cells activate B-cells to become effector plasma-cells that secrete soluble forms of islet-specific immunoglobulin (or autoantibodies) that bind to autoantigens [20].

Identification of novel autoantigenic targets determined by both CD8^+^ and CD4^+^ T-cells is relatively imperative to the theoretical and experimental understanding of the immunologic processes which contribute to a cytotoxic humoral and/or cell-mediated anamnestic response to the destruction of pancreatic islets. Interest in recent immunologic response serology in T1D resulted in the identification of four major molecularly characterized islet specific autoantigens as immunological markers of disease progression: the secreted hormone insulin; the Mr 65,000 isoform of glutamate decarboxylase or glutamic acid decarboxylase (GAD65); islet protein tyrosine phosphatase-like molecule (IA-2) or islet cell autoantigen 512 (ICA512); and the novel T1D autoimmunity target zinc transporter 8 (ZnT8) [5], [21]–[31].

Recent studies [22] have shown that cytoplasmic islet cell antibodies (ICA), a conventional autoantibody marker for T1D, are identified in approximately 85% of children. In addition, islet antigenic determinants augmenting the risk of T1D include a previously identified protein tyrosine phosphatase-like molecule IA-2 [24], which incorporates the intracellular fragment of IA-2 containing the immunodominant epitope 137–143, and a recently specified fragment within the extracellular domain of this protein. Reliable fluid phase radioimmunoassays have been used [5], [27]–[29] to determine the frequency of IA-2 (ICA512) and GAD65 autoantibodies, and thus identify autoimmunity amongst patients with other organ and non-organ-specific autoimmune diseases.

To conclude whether collections of autoantibodies, including those reactive to IA-2 autoantigen(s), may predict T1D, Pietropaolo and colleagues proposed in recent experiments [5] that autoantibodies against a combination of extracellular domain of IA-2 and/or conventional autoimmunity markers can distinguish between rapid and slow progressors of T1D. It has been shown that serum samples from first degree relative of T1D patients that tested positive for autoantibodies reactive to full length IA-2 (IA-FL) and GAD65 but negative for the IA-2 intracellular protein constructs (ICA512bdc) harbored 100% cumulative risk of developing the disease within 11 years of follow up. In contrast, the presence of at least two conventional autoimmunity markers presented a cumulative risk of 58% after 10 years of follow-up and 74% after 15 years of follow-up [32]. The group of relatives of T1D proband with a high cumulative risk is termed rapid progressors, whereas the second group who lack serological responses against full-length IA-2 including the extracellular domain is termed slow progressors.

Recent findings with regards to T1D in animal models [14] show that it is possible to predict the progression of the disease and thus provide an anabolic window of opportunity to hinder the autoimmune disorder, but there is still deficiency in proven therapies for preventive care in humans [33]. The ability to detect specific anti-islet autoantibodies provides the foundation for developing and testing these preventive therapies. In addition, development of fluid phase radioimmunoassays utilizing recombinant human proteins in screening individuals for GAD65 and ICA512 (IA-2) autoantibodies is semiquantitative and to some extent difficult to standardize. Research work on studying the subtle interplay between islet autoantibodies of four primary molecularly characterized autoantigens and T-cell proliferation have also been impeded by the scattering identification of subjects at high risk of developing clinical T1D [34], and therefore, requires an alternative approach. For this purpose, we develop here a dynamic model of T1D: first by including one clone of pathogenic (CD8^+^ and/or CD4^+^) T-cells in combination with B lymphocytes to analyze the interaction between a broad spectrum of autoreactive T-cell avidity and autoantigen-specific autoantibodies in predicting the time interval of T1D disease onset; second by extending the model to incorporate two clones of T-cells, each of which is reactive a given autoantigen and comprised of high and low avidity subclones, together with islet specific autoreactive B-cells and autoantibody-secreting plasma-cells.

The proposed research framework for the two-clone model addresses the level of risk associated with each copy (clone) of autoreactive effector T-cells in terms of uncertainty and variability in their ability to kill 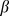-cells [35], by analyzing regimes of model parameters which distinguish between rapid and slow progressors of clinical T1D. Furthermore, due to the nonlinear dynamics of the model and the nature of immunological processes involved in the slow demolition of 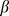-cell population, the model also provides a mechanistic explanation to the phenomenon of relapse-remission occurring during the development of T1D [36] and examines its implications on predicting the disease. Experimental evidence of this phenomenon has been observed, for example, in NOD mice exhibiting periodic waves in the number of effector T-cells before disease onset [37], [38] and in T1D patients that received islet transplants exhibiting fluctuations in the percentage of GAD tetramer-positive T-cells in the CD4^+^ T-cell population from the time of hyperglycemia recurrence [39]. Marée et al. [40] addressing the dynamics of effector T-cells and autoantigenic peptides speculated that sustained elevation in the level of autoantigens, expressed as pMHC complexes that results from 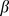-cell apoptosis (or programmed cell death) [41], could be a plausible explanation in generating recurrent waves of autoreactive T-cells. Established upon a set of parameter values and population sizes, our primary objective in this work is to consider the effect of autoreactive T-cell avidity in connection with its killing efficacy in inducing such cyclical fluctuations in effector T-cells, and examine how this could lead to early diagnosis of the disease in high-risk individuals. We also attempt to present the relevant immunological processes affecting the stability of immunity and tolerance in the study of the disease.

## Results

### Dynamics of the One-Clone Model

**Figure 1:**
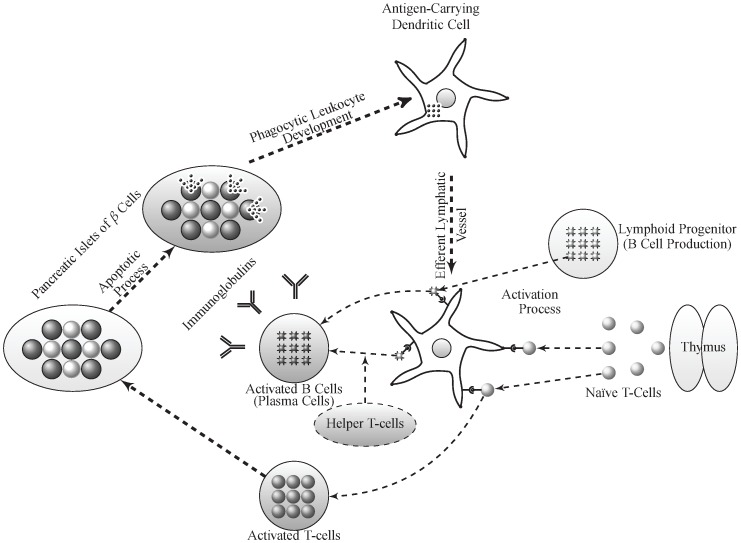
Model diagram of T1D disease progression showing the subpopulations of immune cells implicated in the disease and the transitions between them. Reduced expression of self-antigen(s) in the thymus or extra-thymic lymphoid organs may predispose to T1D and autoimmunity by inducing T-cell positive selection [32]. Nave T-cells leaving the thymus are activated into effector T-cells in the lymph nodes by APCs that express islet-specific autoantigens via TCR interaction with pMHC complexes. These autoreactive T-cells then infiltrate the islets and cause 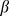-cell destruction at a relatively slow rate. Protein fragments from dead 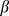-cells are subsequently taken up by APCs for processing and pMHC production, leading to epitope spreading and further amplification of autoreactive T-cell response. B-cell activation into autoantibody-secreting plasma-cells is induced by helper T-cells and APCs via similar mechanism.

According to the Models and Methods Section and Fig. 1, the revised full one-clone model, described by Eqns. (4)–(9) (or the scaled model (S1.13)–(S1.18)), incorporates the Hill function 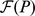, given by Eqn. (S1.1), in the maturation of B-cells into plasma-cells, and includes T-cell killing efficacy 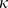 in the expression of pMHC production, two new more physiological features that were ignored in the original one-clone model introduced in [34]. The inclusion of 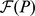 in B-cell maturation also provides us with the necessary means to examine the correlation between T-cell avidity and autoantibody binding affinity (or B-cell avidity) through the parameter 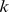 (or 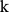 for the scaled model).

Using a combination of stability theory and numerical simulations, we demonstrate in Appendix S1.1 that the revised full one-clone model preserves all the properties of the original one-clone model, including transient bistability and model responses to variations in T-cell avidity and killing efficacy. In particular, in Appendix S1.1, we show that the revised full model can be nondimensionalized, using the substitutions in Eqns. (S1.12), and reduced in size to a scaled two-variable model, consisting of autoreactive T-cells 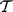 and autoantigen expression level on APCs 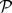 (see Eqns. (S1.19)–(S1.20)), by decoupling the B- and plasma-cell equations from the rest of the model and setting 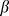-cell number to a constant (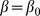, or 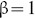), due to its homeostatic mechanisms. Our analysis of the (scaled) reduced model in Appendix S1.1 reveals that it possesses at most three steady states, depending on the value of the parametric quantity 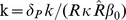. One steady state, the disease-free (healthy) state 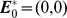 with no elevation in 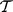, is always present and stable, whereas the other two steady states, a stable autoimmune state 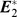 (with elevated level of 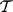) and a saddle point 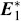, are present whenever
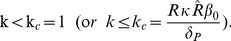
(1)


It is then shown that these two latter steady states 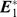 and 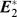 merge at a saddle node bifurcation point when 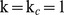 (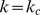) and disappear for 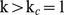 (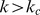). Using Dulac's criterion, we also establish that this (scaled) reduced model will never exhibit any non-negative periodic fluctuations in the number of T-cells (i.e., no periodic orbits in the first quadrant of the 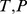-space).

We conclude at the end of Appendix S1.1 that, in the case of the (scaled) full model, the steady states 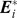 correspond to the steady states 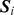, 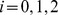, where 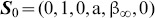 (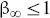) is the disease-free (healthy) state and 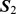 is a transient (quasi-stable) autoimmune state (with temporary elevation in the levels of autoreactive T-cells, B-cells, plasma-cells and autoantibodies). We then establish that 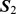 is quasi-stable because of the steady decline in the number of 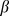-cells (quantified by the scaled variable 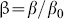) during the course of the autoimmune response. By plotting the heat-maps of 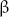 at steady state in Fig. S1.2(A1), and the scaled level of autoantibodies 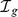 at the following time points: six months after the start of the autoimmune attack, at disease onset for those that develop the disease and at steady state in Fig. S1.2(B1-B3), respectively, using the (scaled) full model and the ranges 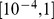 and 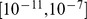 (day cell)^−1^ for 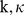, quantifying T-cell avidity and killing efficacy, respectively, we verify that model outcomes are identical to those observed in [34]. In fact, the classifications of high risk subjects in the 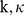-parameter regime to four categories of healthy/diabetic versus autoantibody-positive/negative groups identified here (i.e., rapid and slow progressors) remain the same as those obtained in [34].

Because pMHC-dependent T-cell activation dose response curves have been fitted to a Hill function 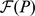, given by Eqn. (S1.27), with a Hill coefficient 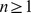 [18], [34], [42], the heat-maps in Fig. S1.2(A2-A3) have been generated to show that increasing the Hill coefficient to 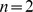 or 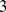, respectively, does not significantly alter the various regimes of behavior obtained in panel (A1) when 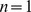, except for increasing the width of the red band on top of each panel. This increase indicates that an ineffective T-cell response is occurring for large 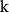, a by-product of both bistability and the steepness of the Hill function at larger 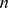, which means that more peptide expression is required for activation. We also plot in panels (C1-C3) the heat maps of the time it takes for the autoantibodies to reach certain detectability levels, given in terms of the scaled quantity 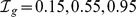, respectively, in response to variations in the two parameters 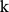 and 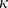 within the ranges specified above. Our results reveal that it takes at most 200 days for 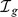 to reach these specific levels, suggesting that seroconversion is quite fast in these high risk subjects.

These results are further illustrated in Fig. S1.3 by examining the 30-year time evolution of the (scaled) full model when 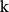 is varied within the range 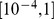 and 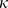 is given specific values within its range 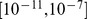 (day cell)^−1^. The heat-maps produced show that increasing 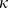 from 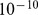 (A1–C1) to 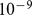 (A2–C2), 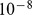 (A3–C3), and 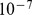 (A4–C4) (day cell)^−1^, leads to a decrease in the length of the time period (shown in red) within which T-cell 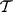 (A1–A4) and autoantibody 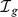 (B1–B4) levels are elevated, accompanied by an increasingly more prominent 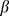-cell destruction, quantified by a gradual time-dependent decrease in 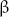 (C1–C4) (shown in blue). The transient bistability exhibited by this model (see Appendix S1.1 and [43] for more details) is the underpinning cause of such behavior. It drives solutions to initially approach the autoimmune state 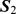, leading to an increase in 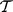 and 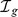, but eventually decline to the “disease-free” state 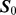 when 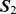 disappears (the disease-free state here is no longer a healthy state). The rise and decline in 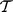 and 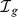 in this case create these “waves” that become even narrower as 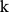 increases towards the threshold for bistability at 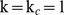, especially for larger values of 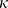, as shown in the bottom panels of Fig. S1.3.

**Figure 2:**
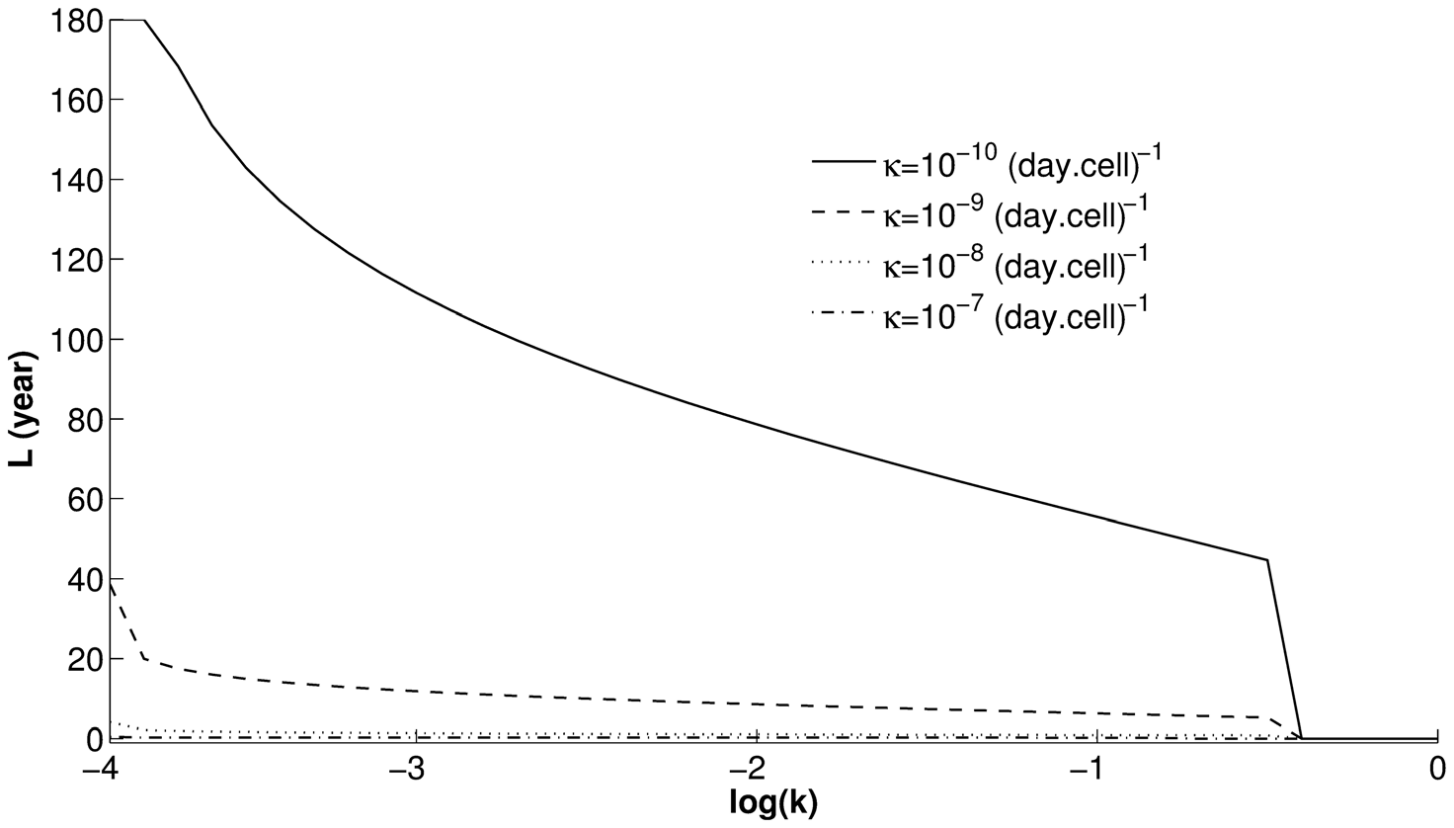
Dependence of T-cell waves, caused by the transience of the autoimmune state 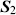, on T-cell avidity and killing efficacy. The duration of T-cell waves 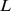 for 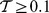 are plotted as a function of the parametric quantity 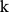, representing T-cell avidity, at the following values of T-cell killing efficacies 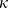: 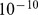 (solid line), 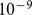 (dashed line), 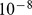 (dotted line) and 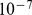 (dashed-dotted line) (day cell)^−1^. These curves are all steadily decreasing functions of 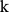 and vanish at 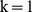 (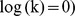.

To capture the effect of 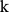 and 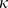 on the width of these T-cell waves, we plot in Fig. 2 the total time duration 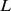 spent above the threshold 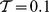 as a function of 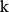 for increasing values of 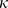. As in Fig. S1.3, we choose the following values of 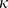: 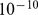 (solid line), 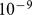 (dashed line), 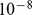 (dotted line) and 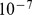 (dashed-dotted line) (day cell)^−1^. The resulting curves in Fig. 2 indicate that (i) the closer the value of 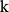 to 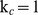 (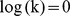), the smaller the 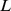; and (ii) the higher the value of 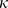, the lower these curves are. In other words, the robustness of (quasi-)stability of the steady state 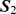 is inversely (directly) correlated with T-cell avidity (killing efficacy). Such behavior is also maintained by the autoantibody waves shown in Fig. S1.3(B1–B4) (results not shown).

Because chronic immunological diseases, such as autoimmune T1D, may be considered clinically as relapsing-remitting disorders [36], we would expect to see cyclic fluctuations in the dynamics of these diseases. In fact, it has been shown in recent work [37] that during the development of T1D in female NOD mice, NRP-V7 (a mimotope of IGRP(206–214)) specific T-cell population exhibits these distinct cycles approximately between 9 and 16 weeks of age prior to the onset of abnormally high level of blood sugar (hyperglycemia). The exact underlying mechanism that control this phenomenon is yet to be discovered experimentally and/or predicted using mathematical modeling. As demonstrated analytically and numerically in this section, the (scaled) one-clone model does not exhibit such cyclic fluctuations due to, as we shall demonstrate in the next section, the lack of cross-clonal competition between T-cells. In other words, to observe the relapse-remission phenomenon and to uncover the immunological processes controlling it, it is essential to expand the study to multi-clone models that take into account competing clones of T-cells.

### Dynamics of the Two-Clone Model

As suggested in the previous section, developing a two-clone model, consisting of two-autoantigenic specificities, is necessary to account for intra- and cross-(sub)clonal competition required for capturing physiological features of T1D that are otherwise not realized by the (scaled) one-clone model. In view of the scheme in Fig.1 and Eqns. (10) – (15), the new model is comprised of four T-cell subclones 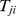, 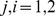, where 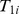 are reactive to 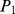 autoantigen and 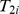 are reactive to the 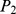 autoantigen. The avidities of these four T-cell subclones, quantified by the parameters 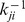, as well as their killing efficacies, are assumed to satisfy the inequalities 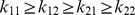 and 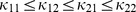 (see Models and Methods Section for more details). Furthermore, as a result of considering two autoantigenic specificities, we expect to have two clones of B-cells 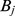, of plasma-cells 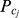 and two islet-specific autoantibodies 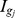, 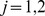, in the model. The 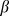-cell destruction induced by the four T-cell subclones, on the other hand, is assumed to be governed by the function 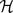 defined in Eqn. (17).

The scaling of this two-clone model in Appendix S1.2 generates fewer parameters and dimensionless variables (see Eqns. (S1.35) – (S1.40)). The new parametric quantities
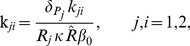
representing T-cell avidity of the four subclones 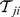, are assumed to obey the same inequality the original avidity parameters satisfied; namely, that
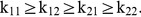
(2)


#### Avidities and disease progression

We begin our analysis of the (scaled) two-clone model by simulating the time evolution of Eqns. (S1.35) – (S1.40) for 30 years. The simulations are performed by varying the avidity of the T-cell subclones one at a time (i.e., varying one avidity and leaving the remaining ones unchanged) within the following ranges:
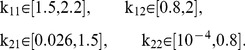
(3)


**Figure 3:**
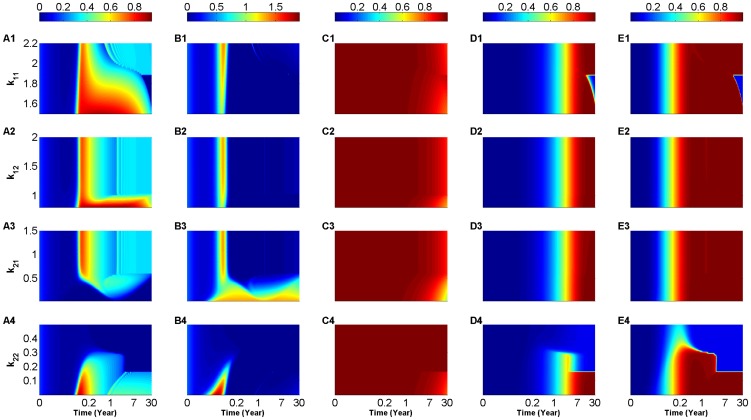
Heat-map simulations of the (scaled) two-clone model (S1.35)–(S1.40) for a low value of T-cell killing efficacy 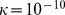 (day cell)^−1^. The 30-year time evolution of the scaled level of 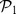-autoreactive T-cells: 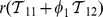 (A1–A4), 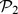-autoreactive T-cells: 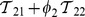 (B1-B4), 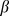-cells: 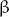 (C1–C4), 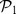-reactive autoantibodies: 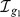 (D1–D4) and 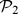-reactive autoantibodies: 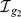 (E1–E4). The colors in these panels represent the levels of these quantities according to the color-bars on top of each column. The simulations are performed over the following ranges of T-cell avidities: 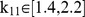 (A1–E1), 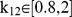 (A2–E2) 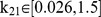 (A3–E3) and 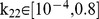 (A4–E4), where inequality (2) is satisfied. Notice here that panels (A1–A4) and (B1–B4) also depict 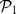
- and 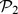-autoreactive T-cell avidity maturation, respectively, during the time-course of the autoimmune attack. Because 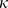 is too small, the magnitude of 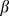-cell loss is minimal, but is sufficient to elevate the level of circulating T-cells.

The resulting heat-maps are displayed in Fig. 3, showing the response of the (scaled) two-clone model to variations in 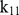 (A1–E1), 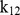 (A2–E2), 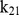 (A3–E3) and 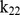 (A4-E4), in accordance with the ranges in (3), while keeping the killing efficacy at low value 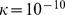 (day cell)^−1^. The different colors in each panel represent the scaled level of effective population sizes of both 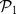-specific T-cells: 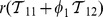 (A1–A4) and 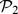-specific T-cells: 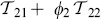 (B1–B4) (which are both measures of avidity maturation), 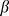-cells: 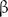 (C1–C4), and 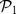-specific: 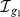 (D1–D4) and 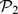-specific: 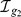 (E1–E4) autoantibodies, according to the color bars on top of each column (with red representing high and blue representing low levels of these quantities). Analogous to the (scaled) one-clone model, the T-cell waves observed in panels (A1–A4) and especially in (B1–B4) for certain parameter ranges within those listed in (3), are induced by several continuously-changing quasi-stable (transient) steady states in this high-dimensional system (see next subsection for more details). The duration of these waves is, however, significantly reduced in length when compared to those observed in the simulations of the (scaled) one-clone model of Fig. S1.3, because of (intra- and cross-clonal) competition between the four subclones of T-cells in the (scaled) two clone-model. In fact, for low values of 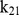 (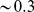), within its defined range in (3), we observe two successive waves in the level of 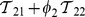 in Fig. 3(B3), resulting from trajectories switching from one quasi-stable steady state to another during the course of the autoimmune attack. [Note that these successive waves are also exhibited in panels (A1–A4) for certain values of 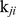.] Furthermore, according to panels (A4) and (B4), these waves disappear completely when the default value of 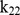 (see Table S1.2 in Appendix S1.2) is increased at least 4-fold, obliterating the two autoantigen specific T-cell clones. Because 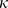 is very small here, the scaled level of 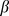-cells 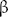 in panels (C1–C4) is largely left intact even after 30 years of follow up despite the presence of T-cell waves.

The time evolution of the scaled level of circulating autoantibodies shown in panels (D1–D4) and (E1–E4) demonstrates that 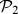-specific autoantibodies, associated with the two higher avidity T-cell subclones 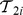, 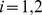, appear first 1–2 months after the start of the autoimmune attack followed by 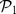-specific autoantibodies that appear 10 months later. Aside from 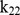, changing the values of 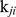, 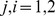, one at a time within the ranges specified in (3), has no effect on such outcomes provided that inequality (2) is satisfied. In the case of 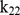, increasing its value causes the two types of autoantibodies to exhibit no or negligible elevation in their levels (see panels (D4) and (E4)), an expected outcome in view of panels (A4) and (B4) showing no elevation in T-cells at higher values of 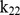. The consecutive appearance of the two autoantibodies within at least 8 months of each other is consistent with experimental observations and is in agreement with our hypothesis suggesting that the higher the avidity of the T-cell (sub)clone in circulation, the earlier the release of cognate autoantibodies from B- and plasma-cells. As for the autoantibodies that correspond to lower avidity T-cell (sub)clones, they appear later, making the total level of autoantibodies exhibit a step-like profile with respect to time. It is important to point out here that the low value of 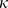 and the insignificant loss of 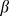 cells in panels (C1–C4) make the outcomes observed in Fig. 3 correspond to slow progressors that screen positive to at least two (conventional) autoantibodies, such as GAD and the intracellular domain of IA-2, but never develop the disease.

**Figure 4:**
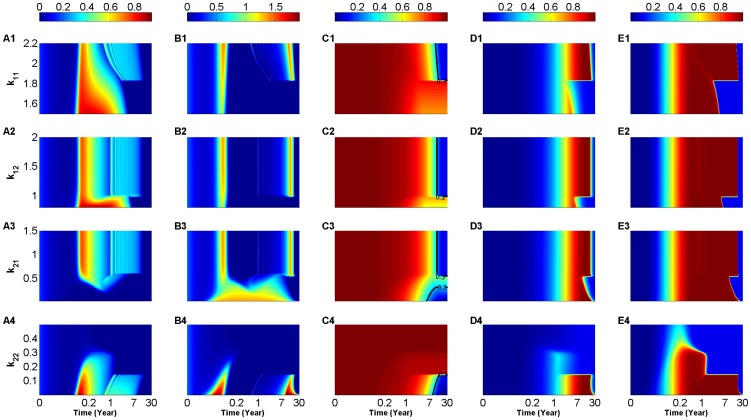
Heat-map simulations of the (scaled) two-clone model (S1.35)–(S1.40) for an intermediate value of T-cell killing efficacy 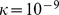 (day cell)^−1^. The 30-year time evolution of the scaled level of 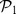-autoreactive T-cells: 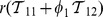 (A1–A4), 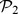-autoreactive T-cells: 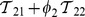 (B1–B4), 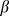-cells: 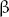 (C1–C4), 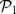-reactive autoantibodies: 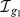 (D1–D4) and 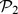-reactive autoantibodies: 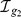 (E1–E4). The colors in these panels represent the levels of these quantities according to the color-bars on top of each column, whereas the black lines in panels (C1–C4) represent the 30% threshold of surviving 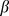-cells (0.3-critical threshold). The simulations are performed over the following ranges of T-cell avidities: 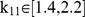 (A1–E1), 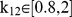 (A2–E2) 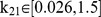 (A3–E3) and 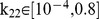 (A4–E4), where inequality (2) is satisfied. Increasing the value of 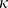 induces oscillations in the level of T-cells and an apparent disease-causing loss in 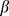-cell level within 15 years from the start of the autoimmune attack.

Notice that for high values of 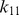, 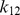 and 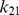 within their respective ranges in (3), the (scaled) two-clone model in Fig. 3(A1–A3) and (B1–B3), respectively, also exhibit these repetitive bands or cyclic fluctuations (to be distinguished from the successive waves discussed earlier) in the two scaled levels of effective T-cell populations. These cyclic fluctuations become even more pronounced when 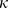 is increased to 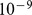 (day cell)^−1^ in Fig. 4, where the 30-year time evolution of the scaled levels of effective population sizes of both 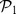-specific T-cells: 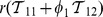 (A1–A4) and 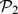-specific T-cells: 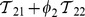 (B1–B4) are plotted as heat-maps with respect to 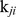 as before. The sudden appearance and termination of these cyclic fluctuations are caused by the presence of two Hopf bifurcation points, details of which are discussed in the next subsection. The three other model features observed in Fig. 3 remain predominantly the same in Fig. 4 when 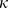 is larger, including: the two successive waves that appear at low 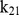 (B3); the depletion of the four T-cell subclones for large 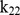 values (A4, B4); and the appearance of the 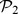-specific autoantibodies (E1–E4) 10 months before the 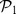-specific autoantibodies (D1–D4) (with the former corresponding to the higher avidity T-cell subclones 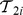, 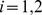).

The larger value of 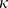 in Fig. 4, however, leads to an increase in the level of 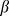-cell destruction, evidenced by the change in color from dark red to blue in panels (C1–C4) for certain values of 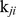 within the ranges listed in (3). By defining the clinical onset of T1D as the time when only 30% of the scaled level of 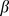-cells is left (called the 0.3-critical threshold and highlighted by the black lines in Fig. 4(C1–C4)), we can see that in most cases, the scaled level of 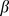-cells decline below the 0.3-critical threshold within 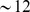 years of the autoimmune attack. The most intriguing result in these panels is the presence of “safe” windows, within which the scaled level of 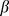-cells never crosses the 0.3-critical threshold for clinical T1D, as indicated by the red and yellow regimes close to the right edge of panels (C1–C4). This suggests that it is possible to either increase the avidities of 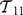-, 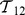
- or 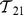-subclones (C1–C3) or decrease the avidities of 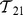
- or 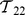-subclones (C3, C4) to land into parameter regimes in which enough repertoire of 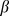-cells survives the autoimmune attack. In light of the analysis of the (scaled) one-clone model in Appendix S1.1, we may conclude that these “safe” windows arise either from (i) solution trajectories initially approaching one of the quasi-stable (transient) autoimmune-states of the (scaled) two-clone model, but eventually propagating towards the stable disease-free state (which is no longer a healthy state because of some 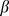-cell loss) as in (C1, C2 and C3); or from (ii) solution trajectories propagating close to the stable manifold of a transient saddle point of the model as in (C3). In the former case, the model predicts that one can clinically manipulate the average avidity of one particular subclone of T-cells (by perhaps injecting a dose of higher avidity T-cells with the same autoantigenic specificity) to drive the immune response into parameter regimes that are less destructive to 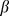-cells and thus less prone to T1D. Targeting the safe window in (C3), on the other hand, is less feasible clinically because of its sensitivity to perturbations.

It should be mentioned here that increasing the value of 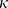 even further to 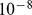 produces results that are almost identical to those observed in Fig. 4, as demonstrated by the heat-maps shown in Fig. S1.4 in Appendix S1.2. In this case, the larger value of 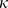 only exacerbating 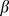-cell destruction and causes faster disease onset by crossing the 0.3-critical threshold (identified by the black lines in Fig. S1.4(C1–C4)) within 1–2 years of the autoimmune attack. We expect to see such outcomes in rapid progressors of T1D, or high risk subjects that test positive to islet-specific (novel) autoantibodies such as those that are reactive to the extracellular domain of IA-2.

#### Relapse-remission in T1D

The underlying mechanism regulating the cyclic fluctuations in the scaled level of effector T-cell populations observed in Figs. 3 and 4 can be examined by using nonlinear bifurcation theory applied on the (scaled) two-clone model described by Eqns. (S1.35)–(S1.40). Performing local bifurcation analysis is an effective way to study the properties of this system and determine how its qualitative dynamic structure changes (sometime unexpectedly) with respect to a smooth variation in the value of one specific parameter (called the bifurcation parameter).

**Figure 5:**
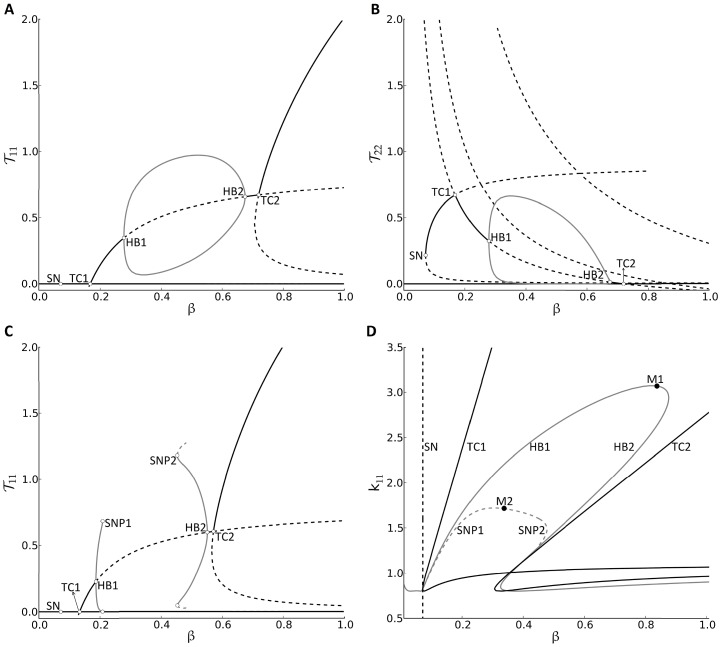
Response of the (scaled) two-clone model (S1.35)–(S1.40) to variations in 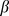-cell number and the avidity of the least avid T-cell subclone 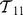. The one-parameter bifurcation of 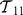 (A) and 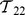 (B) with respect to the parametric quantity 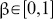, representing the slowly varying scaled level of 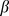-cells, at 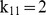 (A–B) and 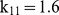 (C), as well as the two-parameter bifurcation of 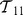 with respect to 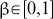 and 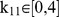 (D). Stable (unstable) steady states in (A–C) are shown as black-solid (black-dashed) lines, and stable (unstable) periodic branches are shown as gray-solid (gray-dashed) lines. The symbols HB1 (TC1) and HB2 (TC2) denote the left and right Hopf (transcritical) bifurcation points, respectively, whereas SN denotes the saddle-node bifurcation point. Furthermore, the symbols SNP1 and SNP2 in (C) denote the left and right saddle node of periodic points. The branches corresponding to the two Hopf (transcritical) bifurcation points are shown as gray- (black-) solid line(s), and the branch(es) corresponding to the saddle node (saddle node of periodic) bifurcation point(s) is (are) represented by black- (gray-)dashed line(s) in the two-parameter bifurcation (D). As shown in (D), the two points HB1 and HB2 meet at the merging point M1 on the gray-solid line and the two points SNP1 and SNP2 meet at the merging point M2 on the gray-dashed line. The HB gray-solid line, SNP gray-dashed line and TC1 black-solid line collide with the SN black-dashed line in (D) at a Bogdanov-Takens bifurcation.

Given that 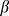-cell loss occurs at a very slow rate (i.e., 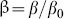 is a slowly changing variable), we may assume that 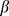 is approximately a constant lying between 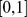. Therefore, it is reasonable to use 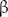 as a bifurcation parameter to understand how the dynamics of the (scaled) two-clone model changes when 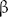 is decreased within 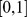. Panels (A) and (B) of Fig. 5 display the bifurcation diagrams of both 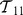 and 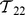 (the least and most avid subclones) with respect to 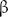, respectively, where 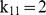 and the remaining parameters of the (scaled) two-clone model are the same as those listed in Table S1.2 in Appendix S1.2.

These panels show that bistability is always exhibited by this model, but it is not always maintained between two distinct steady states; namely, the stable disease-free state with no elevation in T-cells (represented by the horizontal black-solid line close to the 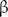-axis) and the stable autoimmune state with an elevation of at least one subclone of T-cells (represented by the black-solid line away from the 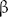-axis). In fact, when the value of 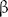 is between the two Hopf bifurcation points HB1 (left) and HB2 (right), where stable periodic branches (gray-solid lines) emerge, the scaled levels of T-cells 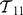 and 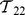 oscillate with amplitudes that are enveloped by these gray-solid periodic branches shown in each panel. Such behavior was absent in the (scaled) one-clone model. Furthermore, these panels indicate that the stable autoimmune state does not necessarily remain qualitatively the same; it gets exchanged from one steady state to another when the value of 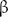 passes through the transcritical bifurcation points TC1 (A, B), lying to the left of HB1, and TC2 (A, B), lying to the right of HB2. The autoimmune state eventually disappears by merging with a saddle point (represented as a black-dashed line in panel (B)) at a saddle-node bifurcation point SN, leaving the disease-free state as the only stable steady state for low 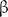.

These bifurcation diagrams illustrate qualitatively how 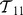 and 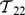 evolve dynamically in response to a gradual decrease in 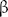, representing 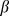-cell decline in T1D. More specifically, they show that it is possible for two subclones to be in circulation (to coexist) during disease progression, as demonstrated by panels (A) and (B), showing 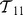 and 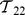 being elevated between the two points TC1 and HB1. They also clarify how the repetitive bands (or cyclic fluctuations) in the effective level of T-cells displayed in Figs. 3, 4 and S1.4 are generated, as well as explain how the successive waves, shown in the same figures, are induced by the gradual displacement (transience) of the stable autoimmune state along the black-solid lines during 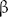-decrease (see Fig. 5(A, B)). These two features of the model, namely, successive waves and cyclic fluctuations, may underlie the relapse-remission phenomenon observed in autoimmune diseases in general and T1D in particular. The longer period of the former versus the more oscillations of the latter are criteria that can be used to determine which one of these two processes is responsible for the clinical onset of relapse-remission in T1D.

We have established in the previous section that the overall behavior of the disease can be improved (through reducing T-cell induced 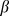-cell damage) by increasing the avidity of the T-cell subclones 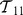 for example. We elucidate such an effect in Fig. 5(C), by decreasing the value of 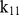 to 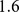, and replotting the bifurcation diagram of 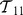 with respect to 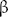. In this case, the stable periodic branches (gray-solid line) emerging from the Hopf points HB1 and HB2 break at a saddle-node of periodic points (SNP1 and SNP2) that give rise to unstable periodic orbits (gray-dashed lines). Between the two points SNP1 and SNP2, bistability is lost and the disease-free state becomes the only attractor. According to this configuration, we expect that the time-dependent decline of 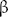 during T1D, from the “healthy” level of 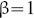, to follow the profile of the stable autoimmune states until it reaches the HB2. Passing through this point triggers oscillations in the four subclones 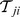, 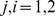, accompanied by a step-wise decline in 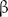, as demonstrated by the simulations in Fig. 4 for example. Unlike the configuration in Fig. 5(A), the step-wise decline in 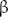 eventually reaches the SNP2 in Fig. 5(C) and the oscillations terminate when the four subclones 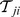 take a sharp drop in their level to the disease-free state. The value of 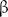 at this point (SNP2) is 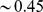 indicating that ∼45% of 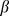-cell repertoire is left intact, which is above the 0.3-critical threshold for the clinical onset of T1D identified in the previous section. Such scenario is significantly better than the one associated with configuration (A), where surviving 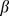-cell level drops below 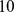
% at the SN. This suggests that it is possible to improve disease outcomes by boosting the avidity of the 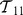-subclone.

The 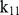-dependent changes in the configurations of 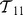
- and 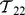-bifurcation diagrams with respect to 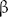 are shown as movies in Appendices S2 and S3, respectively, to illustrate the simultaneous effect of 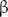-decline and 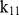-decrease on the model. We summarize these results in the two-parameter bifurcation diagram shown in Fig. 5(D), displaying how the location of the main bifurcation points SN (black-dashed line), TC1, TC2 (black-solid lines), HB1, HB2 (gray-solid line), SNP1 and SNP2 (gray-dashed line) change with respect to the two parametric quantities 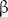 and 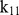. The lines generated enclose regimes that characterize the dynamic behavior of the model and the qualitative properties of the autoimmune state. For example, the HB solid- and SNP dashed-gray lines delineate a bounded regime within which oscillations and bistability are observed with no periodic oscillations outside. Moreover, bistability is always observed to the right of the SN black-dashed line and above the SNP gray-dashed line, alternating between a scenario involving two steady states (autoimmune and disease-free states) and scenario involving a periodic orbit and the disease-free state. Monostability involving the disease-free state, on the other hand, is observed in the regimes to the left of the SN black-dashed line and below the SNP dashed-gray line. These regimes provide an understanding of the rich dynamics the model possesses as well as a platform to target those regimes for healthy outcomes, as established in the previous section.

#### Autoantibody predictability and detectability

In the analysis of the (scaled) one-clone model (see Appendix S1.1), we used the level of autoantibodies to determine the differences between slow and rapid progressors of T1D. To gain more insights into this phenomenon, we turn our attention now to the more physiological (scaled) two-clone model to examine how the the killing efficacy of T-cells and the correlation between T- and B-cell avidities affect both the ability of circulating cognate autoantibodies to predict the timing of disease onset, and the time it takes for them to reach certain detectability levels. To achieve this goal, we evaluate the steady state (or 30-year) response of this model to simultaneous variations in 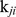, 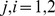, within the ranges specified in (3), subject to inequality (2), and 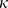 within the range 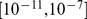 (day cell)^−1^.

**Figure 6:**
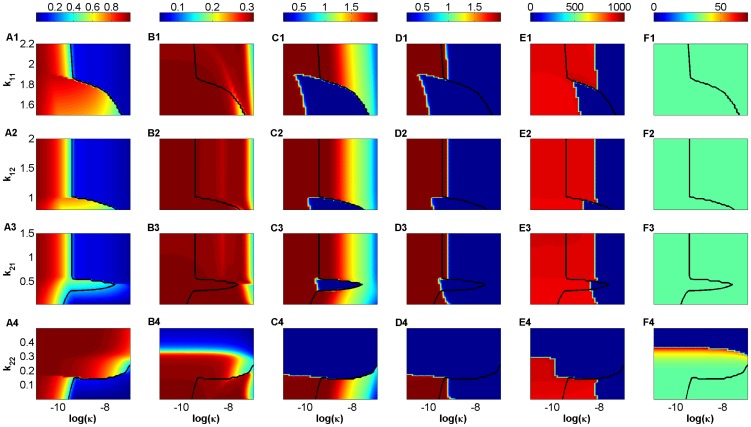
Heat-map simulations of the (scaled) two-clone model (S1.35)–(S1.40) showing the effects of varying T-cell avidity and killing efficacy on autoantibody predictability and detectability. The scaled level of 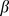-cells at 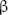 steady state (A1–A4), the total scaled levels of autoreactive autoantibodies 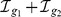 at the three time points: six months after the start of the autoimmune attack (B1–B4), at disease onset for those that develop the disease (C1–C4) and at steady state (D1–D4), and the time duration (in days) for 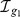 (E1–E4) and 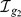 (F1–F4) to reach the detectability level of 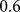, are simulated in response to variations to both T-cell avidity within the ranges 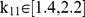 (A1–F1), 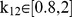 (A2–F2) 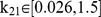 (A3–F3) and 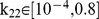 (A4–F4), where inequality (2) is satisfied, and T-cell killing efficacy within the range 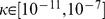 (day cell)^−1^. The black line in each panel represents the 30% threshold of surviving 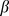-cells (0.3-critical threshold), and the colors represent the levels of these quantities according to the color-bars on top of each column.

The resulting heat-maps displayed in Fig. 6 show that when the killing efficacy 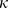 is too small, the steady state level of 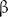 (A1–A4) stays high, regardless of how high the avidities of the four subclones are. Similarly, panel (A4) shows that when 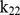 is not too small (i.e., the avidity of 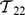 subclone is not too high), then 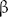 stays also high, independently of 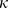, which is in agreement with our previous observations shown in Figs. 3, 4 and S1.4. In the remaining parameter regimes, however, a decrease in 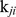, 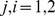, and 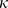 can lead to a steady decline in the steady state level of 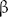, leading eventually to clinical disease for those regimes that lie to the right of the 0.3-critical thresholds shown as black lines in (A1–A4). The shape of these black lines suggest that it is possible to improve disease outcomes significantly by applying very small perturbations to the avidity levels of the 4-subclones (e.g., by increasing the avidity of 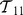 to push the system to the left of the 0.3-critical threshold highlighted in (A1)).

To relate the various observed responses in the steady state level of 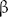 (A1–A4) to the scaled level of autoantibodies 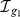 and 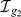, we plot in Fig. 6 the heat-maps of 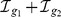 with respect to 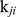 and 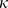 at three different time points: six months after the start of the autoimmune attack (B1–B4), at disease onset for regimes that lie to the right of the black lines (C1–C4) and at steady state (D1–D4). According to these panels, high risk subjects can be classified into (i) slow progressors that test positive to (conventional) autoantibodies their entire life, but never develop the disease (e.g., any point to the left of the 0.3-critical threshold with elevated 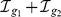); (ii) slow progressors that test positive to (conventional) autoantibodies their entire life and eventually develop the disease at a very slow pace because of low 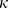 (e.g., any point adjacent to the 0.3-critical threshold on the right); (iii) rapid progressors that test positive most of their lives, except perhaps at steady state, to (novel) autoantibodies and quickly develop the disease because of high 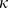 (e.g., regimes that are close to the right edge of (D1–D4)). The scaled level of autoantibodies in the blue regimes of panels (B1–B4), (C1–C4) and (D1–D4) is almost 0.5 which is close to the basal level of autoantibody-release from B-cells (see Table S1.2 in Appendix S1.2). If this level of autoantibody-release is detectable, then even rapid progressors will stay autoantibody positive their entire life.

Notice that in this figure, there are blue regimes in panels (C1–C4) that are surrounded by elevated levels of autoantibodies. The intra- and cross-clonal competition between the four T-cell subclones make the steady state level of 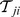 almost near-zero within these blue regimes, and 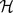, defined by Eqn. (17), to vanish (results not shown), causing such effects on the autoantibody level. In fact, the steady state level of 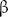 corresponding to these blue regimes is significantly above 0.3 and lies to the right of the black lines shown in panels (A1–A4). Although the long term behavior of four subclones is determined by the disease-free state, the apparent but small decline in 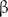 in these regimes can be attributed to the non-zero initial-level assumed for each subclone.

Finally, to determine if avidity or killing efficacy affect how fast the two types of autoantibodies reach their maximum, we plot in panels (E1–E4) and (D1–D4) the heat-maps of the time it takes for 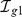 and 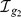, respectively, to reach 0.6-detectability level with respect to 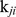 and 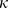. According to these panels, we find that 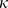 plays no role in the timing of detectability, but avidity does. The latter follows from the faster rise in the scaled level of 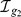, induced by the higher avidity clone 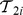, when compared to that of the scaled level of 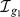, induced by the lower avidity clone 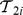, an expected outcome in view of the values of the turnover rates 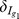 and 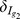 (see Table S1.2 in Appendix S1.2).

## Discussion

The use of competition-based population models of immune and host cells is a very powerful tool to understand the dynamics of autoimmune diseases, in general, and T1D, in particular. Considering the difficulties and challenges associated with performing in-vivo experiments to study the role of immune cells in the onset and progression of these diseases, it is very reasonable to apply these alternative theoretical approaches to achieve similar goals. By focusing on T1D in this paper, we developed series of mathematical models, in the form of ordinary differential equations, that describe the dynamics of T-cells, B-cells, autoantibody-releasing plasma-cells, 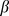-cells, autoantibodies and autoantigens, first by considering one antigenic specificity (one-clone model), followed by a more complex model consisting of two antigenic specificities (two-clone model). Our goal was to determine how the interaction between these different components affect disease outcomes, including the role of avidity and autoantibody-based biomarkers in distinguishing between rapid and slow progressors, as well as the immunological processes controlling relapse-remission phenomenon in T1D patients.

The models developed here were more physiological than those introduced previously [34]. The two major modifications made to the one-clone model were the inclusion of the pMHC-dependent Hill function, described by Eqn. (S1.1), in the expression of B-cell activation, along with the inclusion of T-cell killing efficacy in pMHC production. The former is used to capture the correlation between T-cell and B-cell avidities with the same autoantigenic specificity, while the latter is used to guarantee that the expression level of pMHC on APCs depends on the level of 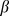-cells killed rather than the total number of surviving 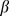-cells. Furthermore, because of limited space in pancreatic lymph nodes and limited number of pMHC-binding sites on APCs, we also modified the two-clone model by including cross-clonal competition between T-cell clones with different autoantigenic specificities. This change allowed us to examine the effects of competition between several T-cell subclones (with different avidities and autoantigenic specificities) on both autoantibody release from their B-cell counterparts and on the progression of T1D under various circumstances.

By applying stability analysis and a reductionist approach, we showed that the one-clone model can exhibit transient bistability between a stable disease-free (healthy) state with no elevation in the level of autoreactive T-cells, and a quasi-stable autoimmune state with temporary elevation in the total number of T-cells accompanied by a decline in the total number of pancreatic 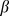-cells. Such transient behavior has been already demonstrated for certain T-cell biomarkers such as FoxP3 and CD25 in effector T-cells for self-regulation [43]. The single waves produced by these transient elevations in the number of T-cells induced similar waves in the level of autoantibodies that lasted longer when T-cell avidity or killing efficacy were decreased. In the two-clone model, successive waves in the effective level of T-cells were observed, as well as cyclic fluctuations (oscillations) emerging from the presence of a transiently stable periodic orbits for specific levels of surviving 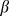-cells. The cross-clonal competition between the different subclones of T-cells in this model was responsible for observing such effects. Given that T1D and other chronic autoimmune diseases are characterized by being relapsing-remitting in nature [36], it is very plausible that these successive waves and/or cyclic fluctuations lie at the root of this phenomenon. Since the period and duration of these two immunological processes differ (with waves having longer periods and less peaks), they can be used as criteria to ascertain which one of them, namely waves or cycles, is constitutively responsible for generating relapse-remission. It should be mentioned here that similar fluctuations in the number of T-cells have been already observed in studies of cancer models describing the alternating phases of tumor progression and regression [44].

The consecutive appearance of autoantibodies of different specificities during epitope spreading in T1D was also captured by the two-clone model. In fact, it was shown that the autoantibodies corresponding to the higher avidity T-cell clone appeared almost 2 years before those corresponding to the lower avidity clone and this behavior was always maintained at all levels of T-cell killing efficacy. Furthermore, the model showed that the rise of autoantibodies to a detectability level of 60% was much faster for the high-affinity autoantibodies than those low-affinity ones, suggesting that the former can be detected earlier in T1D patients than the latter. It was also demonstrated that T-cell killing efficacy played minimal role if any in affecting the timing of autoantibody detectability.

The previous classifications of T1D patients into rapid and slow progressors [34] in terms of the number of surviving 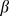-cell number and autoantibody (titer) level measured at different time points was also preserved by the one-clone and two-clone models presented here. Our results showed that slow progressors with elevated levels of (conventional) autoantibodies, such as GAD65, IA-2, insulin and ICA, could either remain healthy (with over 30% of 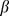-cells still alive) when T-cell killing efficacy is small, or slowly develop the disease when T-cell avidity and killing efficacy were chosen within proper regimes. We also found that rapid progressors with elevated levels of (novel) autoantibodies, such as those against the extracellular domain of IA-2, develop the disease very quickly (because T-cell avidity and killing efficacy are large enough) but may not remain positive their entire life. Such outcomes were consistent with clinically observed data in high risk subjects, suggesting that avidity and killing efficacy are the key factors determining autoantibody-predictability and detectability.

The most striking result predicted by the two-clone model is the ability to improve disease outcomes by manipulating the avidity of the T-cell subclones. More specifically, we discovered that increasing the avidity of the least avid subclones led to an increase in 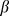-cell survival because of T-cell “depletion” induced by heightened T-cell competition. These results arise from changing the transient dynamics of the model and making the disease-free state a global attractor. The inclusion of more subclones into the model will not only increase its complexity, but also make the interpretation of the results grow exponentially harder. We hypothesize, however, that such multi-clone model will produce repetitive waves that are quantitatively consistent with the number of T-cell clones considered, and preserve the cyclic fluctuations in T-cells in certain parameter regimes. To verify these results, it would be more sensible to assume that the avidity of T-cells is rather a continuum quantity (or independent variable) and develop an integro-differential equation model describing T-cell clonal interactions. This avidity-structured model that we aim to develop will be more physiological and better suited to understand in vivo results.

## Materials and Methods

The complications resulting from the initiation and progression of T1D depend critically on the level of autoreactive and cytotoxic T-lymphocytes. Although, the molecular and cellular dynamics leading to the prevention of disease growth are currently under investigation, the success of these studies require the development of reliable and predictive mathematical models. Islet-specific autoantibodies, which are carried away by the afferent lymph and subsequently the blood, may be considered as biomarkers in determining the risk associated with T1D. Using a series of predictive mathematical models consisting of competing clones of T-cells, our goal here is to study and enhance the abilities of these biomarkers in predicting T1D-risk, as well as decipher the mechanisms underlying certain aspects of this disease. The existing mathematical model in [34] (and relevant models in [45]–[49]) will be further developed to make them more physiologically reasonable as explained below.

### Revised Full One-Clone Model

Based on the scheme in Fig. 1, we initially consider a homogeneous model composed of islet-specific autoreactive T-cells (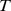), islet specific autoreactive 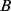 cells (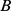), autoantibody-secreting plasma-cells (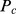), soluble forms of immunoglobulin or autoantibodies (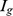) (that can be used as genetic biomarkers to predict T1D), pancreatic 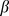-cells (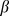), and autoantigens expressed as peptide-MHC (pMHC) complexes (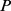) on APCs. In other words, the components of this model are assumed to have one autoantigenic specificity. The differential equation that describes the dynamics of autoreactive (CD8^+^ and CD4^+^) T-cells is expressed as
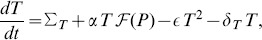
(4)where 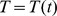 is the population size of T-cells at a given time 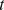, and 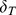 is T-cell turnover. The source term 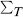, which represents the input of nave T-cells from the thymus, is given by Eqn. S1.5 (see Appendix S1.1 for details). It is assumed to be zero here because having nonzero 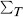 does not alter the dynamics of the model significantly [50]. T-cell replication is described by the term 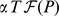, where 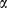 is the replication rate and 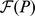 is a Hill function described by Eqn. (S1.1), with a Hill coefficient 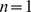 and a pMHC-expression level for half-maximum T-cell activation 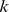. Since 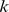 is approximately inversely proportional to T-cell avidity, 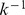 is used as a quantification for T-cell avidity. Intra-clonal competition between T-cells that are reactive to the same autoantigen (to maintain T-cell homeostasis due to limited physical space) is described by the quadratic expression 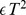.

Islet-specific B-cell activation and maturation into plasma-cells, on the other hand, is regulated by a class of CD4^+^ T-cells, called helper T-cells, and APCs (via B-cell receptor binding with pMHCs, 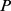), in the presence of various cytokines secreted by T-cells. Applying quasi-steady state approximation on these latter components, namely, the population sizes of helper T-cells and APCs as well as cytokine concentration, we obtain the following differential equations for the dynamics of B- and plasma-cells
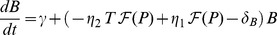
(5)

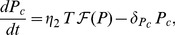
(6)where 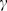 denotes the basal level of B-cell production rate from bone marrow, 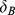 and 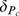 denote turn-over rates of B- and plasma-cells, 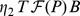 represents B-cell maturation into plasma-cells with a rate 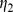, contingent upon pMHC-expression on APCs and cytokine secretion, and 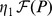 describes B-cell self-renewal by APCs with a rate 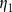. The dependence of B-cell maturation and self-renewal on pMHC expression level on APCs is highlighted by assuming that these two terms are proportional to 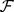. This new modification in the model relative to the one presented in [34] makes the model more physiological and better at reflecting the correlation between T- and B-cell avidities (i.e., T-cell avidity and autoantibody binding affinity).

One of the principal functions of B-cells in circulation is to make autoantibodies against islet-specific autoantigens. They also play a role in antigen presentation, and in expanding into memory B-cells after activation by APCs loaded with pMHCs. However, here we assume that the activation of autoreactive T-cells is mostly carried out by dendritic cells (DCs), a group of myeloid cells that are resident in the body's tissues and have a distinctive star-shaped morphology [51]. The unique function of DCs residing in the infiltrated islets is to work as cellular carriers to stimulate the adaptive immune response.

Autoantibody-secreting plasma-cells are critical immune effector cells that are mostly dedicated to secreting antigen-specific immunoglobulin (or autoantibodies) in the circulation. We assume here that the vast majority of circulating autoantibodies is produced by plasma-cells with a very small fraction from B-cells [34], and that their production is proportional to populations sizes of these two types of immune cells. Based on these assumptions, we conclude that the equation for islet-specific autoantibodies is give by
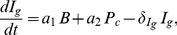
(7)where 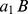 and 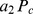 represent autoantibody release by B-cells and (mostly) plasma-cells, respectively, with 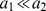, and 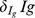 represents autoantibody degradation.

Supported by several experimental evidence, we expect that during T1D progression, the population of insulin-secreting 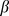-cells to experience loss due to (CD8^+^ and CD4^+^) T-cell induced apoptosis. Autoantigen uptake by APCs (or DCs), from phagocytosed dead 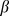-cells, and the production of pMHCs initiate T- and B-cell activation (see Fig. 1). Although there is evidence for B-cell involvement in the destruction of 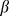-cells during T1D [52], such effect appears not to be as compelling as that of T-cells; therefore, we ignore such effect in our analysis. By focusing only on one clone of T-cells reactive to one given autoantigen, we can, based on the scheme of Fig. 1 and the above assumptions, express 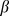-cell dynamics by the following equation
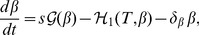
(8)where 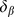 is the turnover rate of 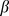-cells per day and 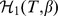 (see Eqn. (S1.3) in Appendix S1.1) describes 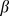-cell killing by T-cells and harmful cytokines with an effective killing rate 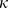 (expected to remain approximately constant throughout disease progression within the range 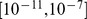 (day cell)^−1^ [34], [38], [50]). 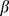-cell neogenesis and replication are both lumped up in the term 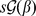 which is a Hill function (see (S1.4) in Appendix S1.1) with maximal regeneration rate 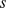 and a saturation parameter 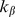 [50]. In all of our simulations, we take 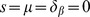 because of either their experimental insignificance or the little effect they exert on the dynamics.

Subsequent to 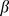-cell apoptosis and phagocytosis by DCs, 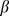-cell proteins are processed by proteasomes into autoantigenic peptides within APCs and expressed as pMHC complexes on their surface. The surface molecules drive T-cell and B-cell activation in the pancreatic lymph nodes. Despite the fact that multiple islet-specific autoantigens are involved in T1D, in the following system, we limit our analysis to only one autoantigenic peptide described by the equation
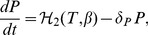
(9)where 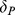 is the degradation rate of pMHC per day and 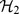 describes pMHC production per T-cell per 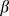-cell (see Eqn. (S1.2) in Appendix S1.1).

### Two-Clone Model

The one-clone model described by Eqns. (4) – (9) is further expanded here by considering multiple T- and B-cell avidities and autoantigenic specificities. Our goal is to illustrate how the increase in complexity (based on physiological assumptions) can capture additional aspects of the disease, particularly the remission relapse phenomenon, that the one-clone model cannot.

We have assumed in the one-clone model a homogeneous structure in which T-cell, B-cell and plasma-cell populations are reactive to only one autoantigenic peptide. In reality, however, several islet-specific autoantigens are involved in T1D, leading to the activation of various clones of T- and B-cells, each with an acquired level of avidity and autoantigenic specificity. We plan here to expand the one-clone model, in a manner similar to what was done in [34], by including the interaction of two clones of T-cells possessing different levels of avidity and autoantigenic specificities with the premise that each one of these clones is comprised of two subclones of high and low avidity T-cells. We also incorporate into the model intra- and cross-(sub)clonal competition between T-cells (arising from the limited physical space available for T-cell expansion and limited number of pMHC binding sites on DCs), a feature that was not previously considered [34]. The inclusion of two autoantigenic specificities implies that B-cells, plasma-cells, islet-specific autoantibodies and peptides expressed as pMHCs on APCs will also consist of two clones or copies. According to this description, the equations describing the two-clone model are given by



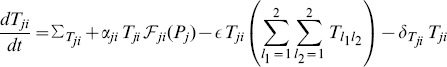
(10)

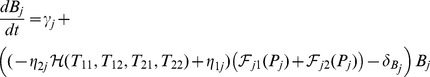
(11)

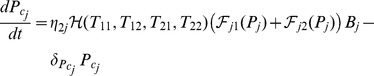
(12)

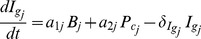
(13)

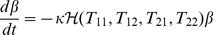
(14)

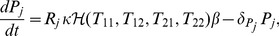
(15)where 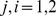,
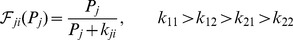
(16)and
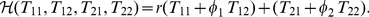
(17)


In this model, the lower avidity subclones 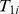 are reactive to 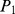 (with 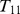 possessing lower avidity than 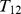) and the higher avidity subclones 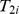 are reactive to 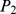 (with 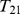 possessing lower avidity than 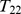). The two clones of B-cells 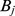 and plasma-cells 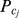 are defined similarly (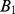 and 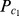 are reactive to 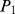, whereas 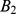 and 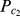 are reactive to 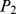, with the former clones possessing lower avidity than the latter clones, respectively). Islet-specific autoantibodies are produced by the two clones of B- and plasma-cells, with 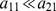 and 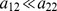. As in the one clone model, T-cell activation is governed by the pMHC expression level on APCs according to the Hill function 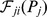 described by Eqn. (16), with a Hill coefficient 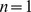 and a pMHC-level for half-maximum T-cell activation 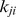. On the other hand, the maturation of B-cells 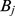 into plasma-cells 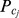 depends on the sum of two of these Hill functions 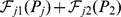. According to this model, the four T-cell subclones are all involved in 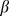-cell destruction via the function 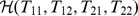, defined by Eqn. (17), but their killing efficacies conform to their avidity levels via the parameters 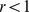 and 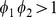. In other words, the killing efficacies of these four subclones satisfy 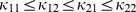, where 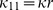, 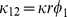, 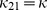 and 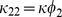 (see Fig. 6 in [34]). Notice here that the quantity 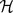 can be used as a measure of total avidity maturation over time for the two distinct and destructive clones of T-cell populations. It can be also used to determine the level of T-cell-induced activation and maturation of B-cells into autoantibody-secreting plasma-cells in the presence of several cytokine and helper T-cells.

The primary component of (CD8^+^ and CD4^+^) T-cell responses to complex autoantigenic peptides is immunodominance, in which subclones of T-cells recognize their cognate ligand and eventually “inhibit/suppress” the proliferation of other T-cell clones engaged with the same APCs. Based on theoretical predictions and experimental findings [53], [54], T-cell populations compete with each other for binding sites on APCs with limited membrane surface area, increasing the competition between T-cells. The effect of cross clonal competition was previously ignored in [34], but here we include such effect to make the model more physiological through the competition term 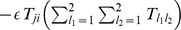, which also includes intra-clonal (direct) competition between T-cells. The remaining terms of the model (10) – (15) are similar to those defined in the one clone model (4) – (9).

### Simulations and Parameter Estimations

Model simulations and bifurcation diagrams were produced using either MATLAB (Mathworks), or the public domain software package AUTO (http://indy.cs.concordia.ca/auto/). The one-clone (Eqns. (4) – (9)) and two-clone (Eqns. (10) – (15)) models were scaled (or nondimensionalized) in Appendices S1.1 and S1.2, respectively, to reduce the number of parameters and to obtain unitless variables. The nondimensionalized (scaled) one-clone (Eqns. (S1.13) – (S1.18)) and two-clone (Eqns. (S1.35) – (S1.40)) models were used to generate all the simulations and bifurcation diagrams shown here and in the Appendices. The (scaled) one-clone model was further analyzed using linear stability theory and reductionist approach in Appendix S1.1. Parameters of the (scaled) one- and two-clone models were previously estimated in [34], but a brief summary of how they were obtained is provided in Appendix S1.1. The parameter values of the (scaled) one- and two-clone models are listed in Table S1.1 and Table S1.2 in Appendix S1, respectively.

## Supporting Information

Appendix S1 **It provides the theoretical analysis used to generate some of the results in the main text.**
(PDF)

Appendix S2
**Movie showing the change in the structure of the bifurcation diagrams of** 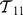 **with respect** 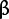 **when** 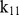 **is changed within the range** 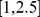 **.**
(MP4)

Appendix S3
**Movie showing the change in the structure of the bifurcation diagrams of** 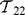 **with respect** 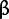 **when** 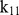 **is changed within the range** 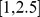 **.**
(MP4)
