# Supplementary material for: Predictive Models of Type 1 Diabetes Progression: Understanding T-Cell Cycles and Their Implications on Autoantibody Release
Source: PLoS One. 2014 Apr 4;9(4):e93326. doi: 10.1371/journal.pone.0093326 (PMC3976292; doi:10.1371/journal.pone.0093326)
Supplement: Appendix S1 — It provides the theoretical analysis used to generate some of the results in the main text. (PDF) [file pone.0093326.s001.pdf]

APPENDIX S1: PREDICTIVE MODELS OF TYPE 1 DIABETES PROGRESSION: UNDERSTANDING T-CELL CYCLES AND THEIR IMPLICATIONS ON AUTOANTIBODY RELEASE

MAJID JABERI-DOURAKI[1], MASSIMO PIETROPAOLO[2] AND ANMAR KHADRA[1]
DEPARTMENT OF PHYSIOLOGY[1]
MCGILL UNIVERSITY, MONTREAL, QC, CANADA H3G 1Y6
LABORATORY OF IMMUNOGENETICS[2]
UNIVERSITY OF MICHIGAN, ANN ARBOR, MI, USA 48105-5714

S1.1. ONE-CLONE MODEL

S1.1.1. **Model Rescaling.** We first start by nondimensionalizing the model (4)–(9) in the main text in order to reduce the number of parameters and simplify the analysis.

Since T-cell activation is regulated by the level of pMHC expression level on APCs, we expect that

$$\frac{d\mathcal{F}}{dP} \geq 0.$$

Based on T-cell dose-response curves to growing level of pMHCs on APCs shown in [1], one can typically describe the function $F(P)$ by a Hill function with a Hill coefficient $n = 1$, given by

$$\mathcal{F}(P) = \frac{P}{P + k}, \tag{S1.1}$$

where k represents the expression level of pMHCs for 50% maximum activation of T-cells. The larger the value of k, the higher the expression level of pMHCs required for T-cell activation, which implies that k^{-1} can be used to quantify T-cell avidity. The empirical determination of the Hill function that approximates T-cell activation (by measuring INF_γ secretion from CD4^+T-cells in response to variations to GAD-expression level on APCs) was also done in [2]. As explained in [3], the value of k for GAD-reactive T-cells was found to lie between [0.02,0.18] μM for high-avidity T-cells [2], but this range was then extended to $[0.1, 9]$ μM for low-avidity T cells. We take advantage of a broad spectrum for k ($\in [0, 20]$ μM) in our analysis of the model (4)–(9) to study the effects of T-cell avidity on disease onset and progression.

The function $\mathcal{H}_1$ in (8) determines β-cell killing by ($\text{CD8}^+$and CD4^+) T-cells occurring at an effective rate κ in the range $[10^{-11}, 10^{-7}]$ (day·cell)$^{-1}$ (which is expected to remain approximately constant throughout disease progression for a given individual [4, 5]). On the other hand, the function $\mathcal{H}_2$ in (9), which describes pMHC production from processing β-cell specific proteins in APCs, is assumed to be proportional to $\mathcal{H}_1(T, \beta)$. In other words,

$$\mathcal{H}_2(T, \beta) = R\,\mathcal{H}_1(T, \beta), \tag{S1.2}$$

where R is the production rate of pMHC per T-cell per β-cell. By using mass-action kinetics to describe the autoimmune attack by $\text{CD8}^+$and CD4^+T-cells and by assuming that β-cell killing is

saturated by β-cell number, we arrive at the following formalism for $\mathcal{H}_1$

$$\mathcal{H}_1(T,\beta) = \kappa T \frac{\beta}{1+\mu\beta}, \tag{S1.3}$$

where μ is the saturation parameter per β-cell for β-cell killing. In some special cases, we may assume that the saturation parameter is identically zero to make $\mathcal{H}_1$ a bilinear function of T-cell and β-cell population sizes. β-cell renewal (via β-cell replication or neogenesis) can be described by (see [4])

$$\mathcal{G}(\beta) = \frac{\beta}{\beta+k_\beta}, \tag{S1.4}$$

where k_β denotes the number of β-cells required for 50% maximal renewal.

In Eqn. (4), the source term Σ_T represents the input of naïve cells from the thymus, which does not necessarily remain constant throughout disease progression. The assumption here is to take the source term Σ_T to satisfy

$$\Sigma_T = \sigma\mathcal{F}(P). \tag{S1.5}$$

By making use of Eqns. (S1.1)–(S1.5), we can give a detailed and explicit mathematical description to the one-clone model (4)–(9) as follows

$$\frac{dT}{dt} = \sigma\frac{P}{P+k} + \alpha T\frac{P}{P+k} - \epsilon T^2 - \delta_T T \tag{S1.6}$$

$$\frac{dB}{dt} = \gamma + \left[(-\eta_2 T + \eta_1)\frac{P}{P+k} - \delta_B\right] B \tag{S1.7}$$

$$\frac{dP_c}{dt} = \eta_2 T\frac{P}{P+k} B - \delta_{P_c} P_c \tag{S1.8}$$

$$\frac{dI_g}{dt} = a_1 B + a_2 P_c - \delta_{I_g} I_g \tag{S1.9}$$

$$\frac{d\beta}{dt} = s\frac{\beta}{\beta+k_\beta} - \kappa T\frac{\beta}{1+\mu\beta} - \delta_\beta\beta \tag{S1.10}$$

$$\frac{dP}{dt} = R\kappa T\frac{\beta}{1+\mu\beta} - \delta_P P, \tag{S1.11}$$

where s is the maximal rate of β-cell renewal per day, whereas δ_β is the turnover (mortality) rate of β-cells per day.

The new model (S1.6)–(S1.11) differ from the one presented in [3] in three main aspects; namely, the presence of a term describing thymus input Σ_T, the dependence of pMHC production rate on κ (T-cell killing efficacy), and the dependence of B-cell activation on the pMHC-dependent Hill function $\mathcal{F}$. These modifications make the current model more physiological, especially the latter one which makes T- and B-cell avidities more closely correlated via the parameter k than previously assumed.

In order to further reduce the complexity of the model or extent of the analysis, we simplify the model (S1.6)–(S1.11) by making the following substitutions

$$\left.\begin{aligned} \mathcal{T} &= \frac{T}{\hat{R}}, & \mathcal{B} &= \frac{\delta_B B}{\gamma}, & \mathcal{P}_c &= \frac{\delta_{P_c} P_c}{\gamma} \\ \mathcal{I}_g &= \frac{\delta_{I_g}\delta_{P_c} I_g}{a_2\gamma}, & \upbeta &= \frac{\beta}{\beta_0}, & \mathcal{P} &= \frac{\delta_P P}{R\kappa\hat{R}\beta_0} \end{aligned}\right\}, \tag{S1.12}$$

where β_0 is the initial number of β-cells right before the autoimmune attack (with the ratio β/β_0 denoted by a non-italicized variable $\upbeta$) and

$$\hat{R} = \frac{\left(\sqrt{\alpha} - \sqrt{\delta_T}\right)^2}{\epsilon}.$$

For simplicity, we assume that $s = \mu = \delta_\beta = 0$. By applying the substitutions introduced in (S1.12) to the model (S1.6)–(S1.11) and using non-italicized font for the newly generated parametric quantities hereafter, we obtain

$$\frac{d\mathcal{T}}{dt} = \upsigma\frac{\mathcal{P}}{\mathcal{P}+\mathrm{k}} + \alpha\mathcal{T}\frac{\mathcal{P}}{\mathcal{P}+\mathrm{k}} - \left(\sqrt{\alpha} - \sqrt{\delta_T}\right)^2\mathcal{T}^2 - \delta_T\mathcal{T} \tag{S1.13}$$

$$\frac{d\mathcal{B}}{dt} = \delta_B + \left[\left(-\upeta_2\mathcal{T} + \eta_1\right)\frac{\mathcal{P}}{\mathcal{P}+\mathrm{k}} - \delta_B\right]\mathcal{B} \tag{S1.14}$$

$$\frac{d\mathcal{P}_c}{dt} = \delta_{P_c}\left(\frac{\upeta_2\mathcal{T}}{\delta_B}\frac{\mathcal{P}}{\mathcal{P}+\mathrm{k}}\mathcal{B} - \mathcal{P}_c\right) \tag{S1.15}$$

$$\frac{d\mathcal{I}_g}{dt} = \delta_{I_g}\left(\mathrm{a}\mathcal{B} + \mathcal{P}_c - \mathcal{I}_g\right) \tag{S1.16}$$

$$\frac{d\upbeta}{dt} = -\kappa\hat{R}\,\upbeta\,\mathcal{T} \tag{S1.17}$$

$$\frac{d\mathcal{P}}{dt} = \delta_P\left(\upbeta\mathcal{T} - \mathcal{P}\right), \tag{S1.18}$$

where the new parametric quantities are given by

$$\upsigma = \frac{\sigma}{\hat{R}}, \quad \mathrm{k} = \frac{\delta_P k}{R\kappa\hat{R}\beta_0}, \quad \upeta_2 = \eta_2\hat{R}, \quad \mathrm{a} = \frac{a_1\delta_{P_c}}{a_2\delta_{\mathcal{B}}}.$$

Thus the number of parameters that need to be estimated have been reduced by introducing these parametric quantities.

S1.1.2. **Parameter Estimation.** For the (scaled) one-clone model (S1.13)–(S1.18), we adopt the parameter values that appeared in [3–10] to perform our simulations. These parameters were estimated using a combination of fit and parsimony. For the definitions, values and units of all these parameters, please see Table S1.1.

In brief, the kinetic parameters associated with B-cell and plasma-cell expansion and turnover were previously determined using in vitro data from [10], whereas those associated with autoantibody release and degradation were determined based on IgG antibody isotype data from [11] and model rescaling (see [3] for more details).

Parameter	Description	Value	Range	Ref.
σ	Influx rate of naïve T-cells from thymus	≈ 0 day^{-1}	–	[4]
α	Expansion rate of T-cells	4 day^{-1}	$[2-20]$	[3,4,6–8]
δ_T	Turnover rate of T-cells	0.1 day^{-1}	$[0.01-0.3]$	[3,4,6–8]
k	pMHC-expression level for 50% maximum activation level of T-cells	0.26	$[0-1]$	[1,3,9]
ϵ	Competition parameter for T-cells	5×10^{-6} (day·cell)$^{-1}$	–	[3,4,6]
δ_B	Turnover rate of B-cells	0.02 day^{-1}	$[0.017-0.02]$	[3,10]
η_1	Expansion rate of B-cells	5.67×10^{-6} day^{-1}	–	[10]
η_2	Maturation rate of B-cells	2.858 day^{-1}	–	[10]
δ_{P_c}	Turnover rate of plasma-cells	0.2 day^{-1}	$[0.116-0.23]$	[3,10]
δ_{I_g}	Degradation rate of autoantibodies	0.034 day^{-1}	$[0.001-0.034]$	[10]
a	Ratio of B-to-plasma autoantibody-release	0.1	–	[10]
κ	T-cell killing efficacy	7×10^{-10} (day·cell)$^{-1}$	$[10^{-11}-10^{-7}]$	[1,3,4]
$\hat{R}$	Ratio of T-cell net growth rate to competition factor	5.6702×10^{5} cells	–	[1,3,4]
δ_P	Degradation rate of autoantigen	0.1 day^{-1}	–	[3–7]

TABLE S1.1. Parameter values of the (scaled) one-clone model (S1.13)–(S1.18).

As for the kinetic parameters of T-cells, β-cells and autoantigen(s), we rely on previously estimated values from [3–8]. These estimates were generated using experimental data available in the literature on T1D. Where relevant, we assign similar values to the parameters listed here.

Because of heterogeneity in the avidities and killing efficacies of the different clones of T1D-specific T-cells (and B-cells), physiologically reasonable ranges for these parameters are instead considered in the simulations shown here. Experimental data from [1, 2] were used to determine such ranges.

S1.1.3. **Phase-Plane Analysis.** In this section, we will study the global dynamics of the one clone model (4)–(9), described in the paper, using steady state and stability analysis. We will show that the full one clone model possesses a stable disease-free (healthy) steady state $S_0 = (0, 1, 0, \mathrm{a}, \beta_\infty, 0)$, with $0.3 \le \beta_\infty \le 1$, that corresponds to healthy individuals, and a transient (quasi-stable) autoimmune state S_2 (with elevated levels of autoreactive T-cells, B-cells, plasma-cells and autoantibodies) that corresponds to type 1 diabetic patients, both separated by a transient interior saddle point S_1. Such behavior is similar to that observed in the one clone model of [3]. In the following, we use a reductionist approach to determine these steady states and analyze their stability properties.

We begin first by noting that due to homeostatic mechanisms, β-cell loss happens at a very slow time scale relative to T-cell dynamics and pMHC processing. Therefore, we may assume that the scaled variable β is roughly a constant, i.e., $\beta = 1$. Furthermore, since $\mathcal{T}$ and $\mathcal{P}$ are decoupled from $\mathcal{B}$, $\mathcal{P}_c$ and $\mathcal{I}_g$, we can ignore the differential equations of these latter variables and only focus on the reduced (scaled one-clone) two-variable subsystem

$$\frac{d\mathcal{T}}{dt} = F(\mathcal{T},\mathcal{P}) := \sigma\frac{\mathcal{P}}{\mathcal{P}+\mathrm{k}} + \alpha\mathcal{T}\frac{\mathcal{P}}{\mathcal{P}+\mathrm{k}} - \left(\sqrt{\alpha} - \sqrt{\delta_T}\right)^2\mathcal{T}^2 - \delta_T\mathcal{T} \tag{S1.19}$$

$$\frac{d\mathcal{P}}{dt} = G(\mathcal{T},\mathcal{P}) := \delta_P\left(\mathcal{T}-\mathcal{P}\right). \tag{S1.20}$$

To investigate the steady state dynamics of the reduced subsystem (S1.19)–(S1.20), we utilize the phase portrait in Fig. S1.1 showing the $\mathcal{T}$- and $\mathcal{P}$-nullclines defined by the equations $\mathcal{T}' = 0$ and $\mathcal{P}' = 0$, respectively. These nullclines subdivide the positive quadrant of the $\mathcal{P}, \mathcal{T}$-plane into regions where $\mathcal{P}'$ and $\mathcal{T}'$ have various signs. Figure S1.1 also shows the vector-field to illustrate the overall flow pattern of solution trajectories of the reduced model. The steady states of the reduced model lies at the intersection of these nullclines. The origin $\boldsymbol{E}_0^* = (0,0)$ is a steady state representing the disease-free state, whereas the other steady states $\boldsymbol{E}^* = (\mathcal{T}^*, \mathcal{P}^*)$, if they exist, must satisfy

$$\mathcal{P}^* = \mathcal{T}^* \tag{S1.21}$$

and

$$\sigma \frac{1}{\mathcal{T}^* + \mathrm{k}} + \alpha \frac{\mathcal{T}^*}{\mathcal{T}^* + \mathrm{k}} = \delta_T + \left(\sqrt{\alpha} - \sqrt{\delta_T}\right)^2 \mathcal{T}^*, \tag{S1.22}$$

where $\mathcal{T}^* \neq 0$. Figure S1.1 shows that when this equation is satisfied, at most two more interior equilibria $\boldsymbol{E}_1^* = (\mathcal{T}_1^*, \mathcal{P}_1^*)$ and $\boldsymbol{E}_2^* = (\mathcal{T}_2^*, \mathcal{P}_2^*)$ must also be present, with the latter representing the autoimmune state. In fact decreasing the value of k gradually shifts the $\mathcal{T}$-nullcline upward and increases the number of intersections between the two nullclines from zero to two. This is illustrated in Fig. S1.1 in which we see either no intersection for $\mathrm{k} > \mathrm{k}_c$ (gray dashed-dotted line), one intersection at $\mathrm{k} = \mathrm{k}_c$ (gray dashed line) and two intersections for $\mathrm{k} < \mathrm{k}_c$ (black solid line), where k_c is the critical value of k at which the two points $\boldsymbol{E}_1^*$ and the autoimmune state $\boldsymbol{E}_2^*$ merge. The value of k_c can be determined by solving for $\mathcal{T}^*$ from (S1.22) as follows

$$\mathcal{T}^*(\mathrm{k}) = \frac{(\alpha - \delta_T - \ell^2\mathrm{k}) \pm \sqrt{(\alpha - \delta_T - \ell^2\mathrm{k})^2 + 4(\sigma - \delta_T\mathrm{k})\ell^2}}{2\ell^2}, \tag{S1.23}$$

where $\ell = \sqrt{\alpha} - \sqrt{\delta_T}$ (taken to be positive). Therefore to obtain one root to Eqn. (S1.23), k_c must satisfy the quadratic equation

$$(\alpha - \delta_T - \ell^2\mathrm{k})^2 + 4(\sigma - \delta_T\mathrm{k})\ell^2 = 0,$$

whose roots are

$$\mathrm{k}_c = \frac{(\alpha + \delta_T) \pm 2\sqrt{\alpha\delta_T - \sigma\ell^2}}{\ell^2}. \tag{S1.24}$$

If we ignore thymus input by setting $\sigma = 0$, we obtain $\mathrm{k}_c = 1$ (or $k_c = R\kappa\hat{R}\beta_0/\delta_P$ for the nonscaled parameter). [The second root $\mathrm{k}_c = [(\sqrt{\alpha} + \sqrt{\delta_T})/(\sqrt{\alpha} - \sqrt{\delta_T})]^2$ is rejected because $\mathcal{T}^*(\mathrm{k}_c) < 0$, unlike the first root for which $\mathcal{T}^*(1) = \sqrt{\delta_T}/\ell > 0$, where $\ell > 0$.]

Because the thymus input does not alter the dynamics of the model significantly [7], we only focus our analysis of the stability properties of all possible steady states hereafter and throughout the paper to the case when $\sigma = 0$. This is done by evaluating the Jacobian matrix of the reduced

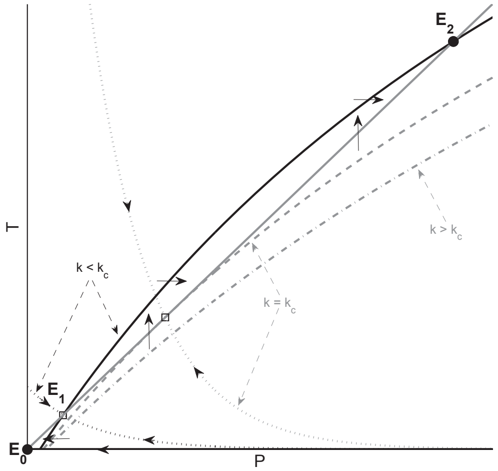

FIGURE S1.1. The phase portrait of the reduced (scaled) one-clone model (S1.19)–(S1.20), with $\sigma = 0$ and $\ell > 0$, displaying the nullclines at different values of k. The $\mathcal{T}$- and $\mathcal{P}$-nullclines are shown as solid black and gray lines, respectively, for $k < k_c = 1$ (the $\mathcal{P}$-axis is also a $\mathcal{T}$-nullcline). Increasing the value of k shifts the $\mathcal{T}$-nullcline downward, becoming first tangential to the $\mathcal{P}$-nullcline at $k = k_c$ (dashed gray line), then lying entirely below the $\mathcal{P}$-nullcline when $k > k_c$ (dashed-dotted gray line). The steady states, lying at the intersection of the $\mathcal{T}$- and $\mathcal{P}$-nullclines, $\boldsymbol{E}_0^*$ and $\boldsymbol{E}_2^*$ (stable nodes) are shown as black dots and $\boldsymbol{E}_1^*$ (saddle) as hollow squares. The stable manifold of the saddle point $\boldsymbol{E}_1^*$ (the black and gray dotted curves) is the separatrix that demarcates the basin of attraction of $\boldsymbol{E}_0^*$ from that of $\boldsymbol{E}_2^*$.

(scaled) one-clone model (S1.19)–(S1.20) at these steady states, as follows

$$
\begin{aligned}
J(\mathcal{T},\mathcal{P}) &= \begin{pmatrix} \dfrac{\partial F(\mathcal{T},\mathcal{P})}{\partial \mathcal{T}} & \dfrac{\partial F(\mathcal{T},\mathcal{P})}{\partial \mathcal{P}} \\ \dfrac{\partial G(\mathcal{T},\mathcal{P})}{\partial \mathcal{T}} & \dfrac{\partial G(\mathcal{T},\mathcal{P})}{\partial \mathcal{P}} \end{pmatrix} \\
&= \begin{pmatrix} \dfrac{\alpha \mathcal{P}}{\mathcal{P}+k} - \delta_T - 2\left(\sqrt{\alpha} - \sqrt{\delta_T}\right)^2 \mathcal{T} & \dfrac{\alpha \mathcal{T}}{\mathcal{P}+k} - \dfrac{\alpha \mathcal{T} \mathcal{P}}{(\mathcal{P}+k)^2} \\ \delta_P & -\delta_P \end{pmatrix}.
\end{aligned} \tag{S1.25}
$$

The eigenvalues of the Jacobian matrix at the disease-free (healthy) state $\boldsymbol{E}_0^* = (0,0)$ are both negative ($\lambda_1 = -\delta_T$ and $\lambda_2 = -\delta_P$), which means that $\boldsymbol{E}_0^*$ is always a stable node. The other two steady states $\boldsymbol{E}_1^*$ and the autoimmune state $\boldsymbol{E}_2^*$, on the other hand, will only exist if $k < k_c = 1$ and $\ell > 0$. Assuming that these two conditions are satisfied and noting that the trace of the Jacobian matrix is always negative, it suffices to show that $\det(J(\boldsymbol{E}_1^*)) < 0$ and $\det(J(\boldsymbol{E}_2^*)) > 0$ to prove that $\boldsymbol{E}_1^*$ is a saddle and $\boldsymbol{E}_2^*$ is a stable node [12]. To achieve this goal, we evaluate the determinant of the Jacobian matrix and determine when it changes sign. Since every non-zero steady state (i.e., those that are not $\boldsymbol{E}_0^* = (0,0)$, when $\sigma = 0$) of the reduced (scaled) one-clone model (S1.19)–(S1.20)

must satisfy (S1.21) and (S1.22), then according to (S1.25), we have

$$\begin{aligned}\det(J(\mathcal{T},\mathcal{P})) &= \delta_P\left(\sqrt{\alpha}-\sqrt{\delta_T}\right)^2\mathcal{T}-\delta_P\left(\frac{\alpha\mathcal{T}}{\mathcal{P}+\mathrm{k}}-\frac{\alpha\mathcal{T}\mathcal{P}}{(\mathcal{P}+\mathrm{k})^2}\right)\\ &= \delta_P\left(-\delta_T+\frac{\alpha\mathcal{T}^2}{(\mathcal{T}+\mathrm{k})^2}\right)\\ &= \delta_P\left(-\delta_T+\frac{\left(\delta_T+\left(\sqrt{\alpha}-\sqrt{\delta_T}\right)^2\mathcal{T}\right)^2}{\alpha}\right).\end{aligned}$$

Thus $\det(J(\mathcal{T},\mathcal{P}))=0$, when $\mathcal{T}=\overline{\mathcal{T}}=\sqrt{\delta_T}/(\sqrt{\alpha}-\sqrt{\delta_T})=\sqrt{\delta_T}/\ell$, which is identical to the value of $\mathcal{T}^*(\mathrm{k}_c=1)$. This means that if $\mathcal{T}>\overline{\mathcal{T}}$, as is the case for the autoimmune state $\boldsymbol{E}_2^*$, then $\det(J(\mathcal{T},\mathcal{P}))>0$ and $\boldsymbol{E}_2^*$ is a stable node, whereas if $\mathcal{T}<\overline{\mathcal{T}}$, as is the case for $\boldsymbol{E}_1^*$, then $\det(J(\mathcal{T},\mathcal{P}))<0$ and $\boldsymbol{E}_1^*$ is a saddle. At $\mathcal{T}=\overline{\mathcal{T}}=\mathcal{T}^*(\mathrm{k}_c=1)$, we have a saddle-node bifurcation as demonstrated in Fig. S1.1.

These latter stability results illustrate the local behavior of the reduced (scaled) one-clone model (S1.19)–(S1.20) near its steady states, when $\sigma=0$. It remains to check if these results are globally valid. In the following analysis, we show that they are indeed global. This is done by establishing first that the solutions of the reduced (scaled) one-clone model, whose initial conditions belong to the space $\mathbb{R}^+\times\mathbb{R}^{\geq 0}$, are bounded and nonnegative, and by taking advantage of Dulac's Criterion to rule out the existence of closed orbits in that space.

Theorem S.1 Let $(\mathcal{T}(t),\mathcal{P}(t))$ be a solution to the (decoupled) reduced (scaled) one-clone model (S1.19)–(S1.20) satisfying $\sigma=0$ and $\ell>0$, with initial condition $(\mathcal{T}(t_0),\mathcal{P}(t_0))\in\mathbb{R}^+\times\mathbb{R}^{\geq 0}$. Then $(\mathcal{T}(t),\mathcal{P}(t))$ is bounded and nonnegative in the state-space $\mathbb{R}^+\times\mathbb{R}^{\geq 0}$. [The result also holds for $\sigma\neq 0$.]

We prove this by applying comparisons method. Since $\mathcal{P}/(\mathcal{P}+\mathrm{k})\leq 1$, we conclude that

$$\frac{d\mathcal{T}}{dt}<\alpha\mathcal{T}-\left(\sqrt{\alpha}-\sqrt{\delta_T}\right)^2\mathcal{T}^2-\delta_T\mathcal{T}.$$

This implies that $\mathcal{T}$ must be bounded by the solutions of the equation

$$\frac{d\widetilde{\mathcal{T}}}{dt}=(\alpha-\delta_T)\widetilde{\mathcal{T}}-\left(\sqrt{\alpha}-\sqrt{\delta_T}\right)^2\widetilde{\mathcal{T}}^2. \tag{S1.26}$$

By analyzing the phase portrait of Eqn. (S1.26), we conclude that

$$\widetilde{\mathcal{T}}\leq \mathrm{C}_1:=\max\left(\widetilde{\mathcal{T}}(0),\frac{\sqrt{\alpha}+\sqrt{\delta_T}}{\ell}\right),$$

where $\widetilde{\mathcal{T}}(0)\geq 0$. It follows that $\mathcal{T}<\mathrm{C}_1$. Substituting this upper bound into Eqn. (S1.20), we get

$$\frac{d\mathcal{P}}{dt}<\delta_P\left(\mathrm{C}_1-\mathcal{P}\right).$$

Applying the same approach used above, we conclude that $\mathcal{P}$ is also bounded by the solutions of the equation

$$\frac{d\widetilde{\mathcal{P}}}{dt} = \delta_P \left(C_1 - \widetilde{\mathcal{P}}\right),$$

where

$$\widetilde{\mathcal{P}} \leq C_2 := \max\left(\widetilde{\mathcal{P}}(0), C_1 + C_2\right)$$

and $\widetilde{\mathcal{P}}(0) \geq 0$. In other words, $\mathcal{P} < C_2$. This proves that $\mathcal{T}(t)$ and $\mathcal{P}(t)$ are both bounded above.

Finally, notice that if $\mathcal{P}(t^*) = 0$ or $\mathcal{T}(t^*) = 0$, for some $t^* \geq t_0$, where $(\mathcal{T}(t_0), \mathcal{P}(t_0)) \in \mathbb{R}^+ \times \mathbb{R}^{\geq 0}$, then, according to Eqns. (S1.19) and (S1.20), we have $\dot{\mathcal{P}}(t^*) \geq 0$ or $\dot{\mathcal{T}}(t^*) = 0$, respectively. This implies that solutions to system (S1.19)–(S1.20), with $\sigma = 0$ and $\ell > 0$, starting from the first quadrant will never leave the quadrant. In other words, $\mathcal{T}(t)$ and $\mathcal{P}(t)$ are both bounded below by zero and that completes the proof.

We now show that no periodic orbits exist using Dulac's Criterion.

Theorem S.2 [Dulac's Criterion, see [13], pp 202] Let $\dot{\mathbf{x}} = \mathrm{f}(\mathbf{x})$ be a continuously differentiable vector field defined on a simply connected subset R of the plane. If there exists a continuously differentiable, real-valued function $\mathrm{g}(\mathbf{x})$ such that $\nabla \cdot \mathrm{g}(\mathbf{x})$ has one sign throughout R, then there are no closed orbits lying entirely in R.

By choosing the continuously differentiable vector field $\mathrm{g}(\mathcal{T}, \mathcal{P}) = 1/\mathcal{T}$ in $\mathbb{R}^+ \times \mathbb{R}^{\geq 0}$, and applying Dulac's Criterion on $\dot{\mathbf{x}} = (\dot{\mathcal{T}}, \dot{\mathcal{P}})$ that satisfies the reduced (scaled) one-clone model (S1.19)–(S1.20) (with $\sigma = 0$ and $\ell > 0$), we obtain

$$\begin{aligned}
\nabla \cdot (\mathrm{g}\dot{\mathbf{x}}) &= \left(\frac{\partial}{\partial \mathcal{T}}, \frac{\partial}{\partial \mathcal{P}}\right) \cdot \left(\frac{1}{\mathcal{T}}\left(\dot{\mathcal{T}}, \dot{\mathcal{P}}\right)\right) \\
&= \left(\frac{\partial}{\partial \mathcal{T}}, \frac{\partial}{\partial \mathcal{P}}\right) \cdot \left[\frac{1}{\mathcal{T}}\left(\alpha \mathcal{T} \frac{\mathcal{P}}{\mathcal{P} + \mathrm{k}} - \left(\sqrt{\alpha} - \sqrt{\delta_T}\right)^2 \mathcal{T}^2 - \delta_T \mathcal{T}\right), \frac{\delta_P}{\mathcal{T}}(\mathcal{T} - \mathcal{P})\right] \\
&= -\left(\sqrt{\alpha} - \sqrt{\delta_T}\right)^2 - \frac{\delta_P}{\mathcal{T}} < 0.
\end{aligned}$$

The control condition $\nabla \cdot (\mathrm{g}\dot{\mathbf{x}})$ is always negative for $\mathcal{T} > 0$ and $\mathcal{P} \geq 0$. As a result, there are no closed orbits lying entirely in the space $(\mathcal{T}, \mathcal{P}) \in \mathbb{R}^+ \times \mathbb{R}^{\geq 0}$. Furthermore, we already know from the previous result that solutions starting from $\mathbb{R}^+ \times \mathbb{R}^{\geq 0}$ will never leave this space. Thus no periodic orbits would lie partially within $\mathbb{R}^+ \times \mathbb{R}^{\geq 0}$. These conclusions guarantee that the local stability properties of the steady states $\boldsymbol{E}_i^*$, $i = 0, 1, 2$, are also global. [Similar result can be obtained for $\sigma \neq 0$.] In this case, when $\mathrm{k} < \mathrm{k}_c$, we have a bistable system with the stable manifold of the saddle point $\boldsymbol{E}_1^*$ (plotted as a black dotted line in Fig. S1.1) is a separatrix that divides the two basins of attraction of $\boldsymbol{E}_0^*$ and $\boldsymbol{E}_2^*$. This configuration remains almost the same when $\mathrm{k} = \mathrm{k}_c$. Here the separatrix (plotted as a gray dotted line) becomes the boundary between the basin of attraction of $\boldsymbol{E}_0^*$ and the basin of attraction of $\boldsymbol{E}_1^* = \boldsymbol{E}_2^*$, which is a half-stable steady state.

It is important to point out that when considering the full model (S1.13)–(S1.18), the steady states $\boldsymbol{E}_i^*$, $i = 0, 1, 2$, automatically translate into the steady states $\boldsymbol{S}_i$ introduced at the beginning of this section. However, due to the fact that $\dot{\beta} < 0$ for $\mathcal{T} > 0$ in Eqn. (S1.17), we expect $\beta(t)$

to be an exponentially decaying function of time, and that the two steady states S_1 and S_2, which both possess elevated levels of T-cells, to be transient steady states, whenever $\mathrm{k} < \mathrm{k}_c$. In other words, solution trajectories that start from the basin of attraction of S_2 will initially approach S_2, but eventually turn towards S_0 when the slope of the hyperplane $\mathcal{T} = \mathcal{P}/\beta$ becomes large enough that the two steady states S_1 and S_2 merge and disappear (i.e., k becomes larger than k_c). Similar behavior was previously observed in [3].

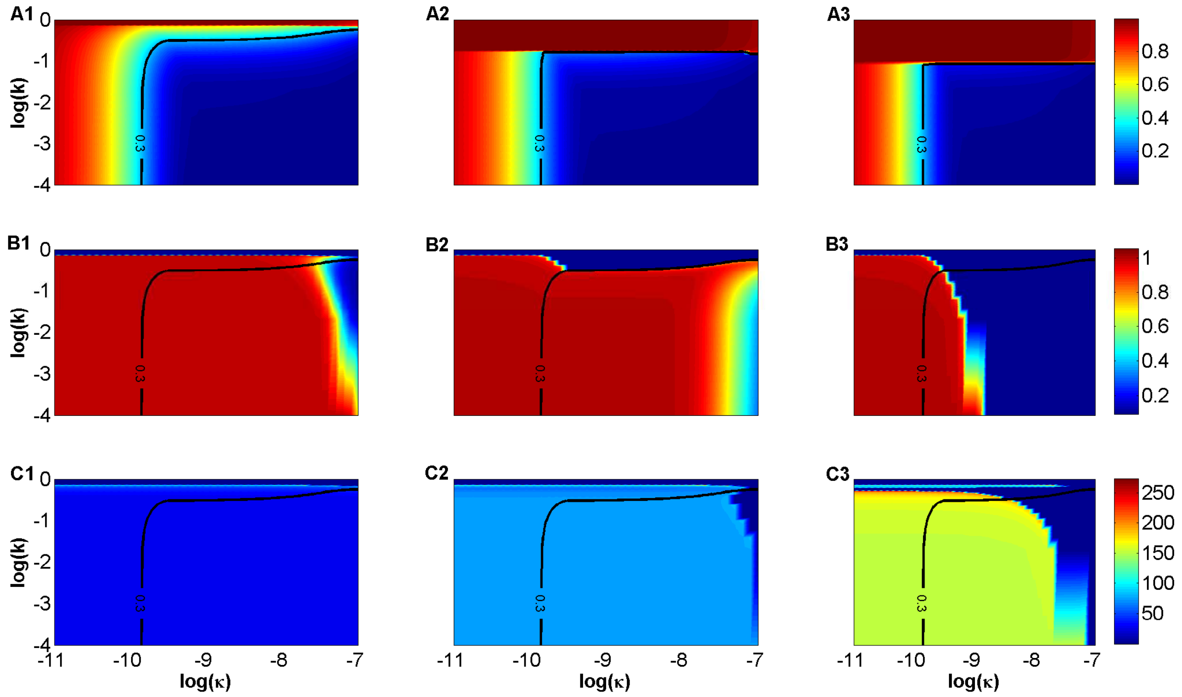

FIGURE S1.2. Heat-maps showing the response of the full (scaled) one clone model (S1.13)–(S1.18) to variations in T-cell avidity (k^{-1}) and killing efficacy (κ) within the ranges $[10^{-4}, 1]$ and $[10^{-11}, 10^{-7}]$ (day·cell)$^{-1}$, respectively. (A1-A3) The steady state level of β-cells (β) for $n = 1$ (A1) $n = 2$ (A2) and $n = 3$ (A3), where n is the Hill coefficient in Eqn. (S1.27), showing the magnitude of β-cell loss in each case. (B1-B3) The autoantibody level $\mathcal{I}_g$ after six months of the autoimmune attack (B1), at the onset of the disease (for those that develop it) (B2) and at steady state (B3). (C1-C3) The time period between the start of the autoimmune attack and when $\mathcal{I}_g$ reaches the following detectability levels: $I_g = 0.15$ (C1), $I_g = 0.55$ (C2) and $I_g = 0.95$ (C3). The black line in each panel represents the 30% threshold of surviving β-cells (0.3-critical threshold). The color-coding in each panel is quantified by the color-bars on the right.

S1.1.4. **Model Simulations.** To illustrate the dynamics of the full (scaled) one-clone model in the presence of all of its components, we simulate system (S1.13)–(S1.18) in response to variations to two key parameters in the model; namely, the reciprocal of T-cell avidity k and T-cell killing efficacy κ (other parameter values are available in Table S1.1). In these simulations, the scaled

variables are used as representatives to the original variables of system (S1.6)–(S1.11). Furthermore, because thymus input is ignored, the initial T-cell level $\mathcal{T}$ is taken to be nonzero but $\ll 1$. Our goal in these simulations is to show that modifying the (scaled) one-clone model, by making B-cell activation depend on k (or k in the nonscaled model (S1.6)–(S1.11)) via the Hill function

$$F(\mathcal{P}) = \frac{\mathcal{P}^n}{\mathcal{P}^n + \mathrm{k}^n}, \tag{S1.27}$$

where $n = 1$ is the Hill coefficient, will produce similar results to those obtained from the one-clone model analyzed in [3] and to uncover other aspects of the model.

Figure S1.2(A1) shows the heat-map of the (scaled) level of β-cells (β) at steady state (i.e., after 30 years of the autoimmune attack) when k and κ are varied within the ranges $[10^{-4}, 1]$ and $[10^{-11}, 10^{-7}]$ (day·cell)$^{-1}$, respectively. We see that the magnitude of β-cell loss (shown as a gradual change in color from red to blue) increases steadily by increasing T-cell avidity and/or its killing efficacy in a manner identical to what have been observed previously in [3]. The presence of the red band on top of each panel demonstrates that if T-cell avidity is too small, then β-cells are safe from T-cell destruction regardless of the level of T-cell killing efficacy κ. Increasing the Hill-coefficient to $n = 2$, in panel (A2), or $n = 3$, in panel (A3), does not alter the heat-map of β significantly. The only noticeable difference we observe is the increase in the width of the red band as n increases. The increase in the steepness of the Hill function, described in (S1.27), for larger n means higher pMHC expression level on APCs is required for T-cell activation and thus wider red bands. The sudden change from red to blue when moving vertically downward across these red bands, however, is caused by the presence of bistable nodes whose basins of attractions are separated by the the stable manifold of the saddle point (the separatrix discussed in the previous section) which generates the boundary of these red bands.

We also observe on the left side of panels (A1-A3) red bands that are less darker than those on top. These red bands demonstrate that most β-cells survive the autoimmune attack whenever T-cell killing efficacy is very small (i.e., when $\kappa \in [10^{-11}, 5 \times 10^{-10}]$ (day·cell)$^{-1}$). For larger values of κ, on the other hand, a more significant decline in β is detected. In fact, increasing the value of κ beyond the critical threshold, highlighted by the thick black lines, labeled 0.3, makes β-cell survival below 30%, indicating clinical manifestation of the disease (through the appearance of T1D-associated symptoms). In the extreme cases identified in the bottom right corner of panels (A1-A3), the survival rate of β-cells is very small due to the effective autoimmune assault dominated by high-avidity, high-killing efficacy T-cells.

Given that B-cell activation also depends on the Hill function described by (S1.27) with $n = 1$, we further characterize the effect of k and κ on the level and survival of circulating autoantibodies. To do so, we plot in panels (B1-B3) the heat-map of $\mathcal{I}_g$ at three successive time points: six months after the inception of the autoimmune attack (B1), at the clinical onset of the disease when the 0.3-threshold is crossed (which applies only to the points to the right of the black line) (B2) and at steady state (30 years after the inception of the autoimmune attack) (B3). Notice that model outcomes here are almost identical to those observed by the one clone model described in [3]. In brief, we observe four possible scenarios: $\mathcal{I}_g$ becomes elevated without reaching diagnostic T1D (to the left of the black line), $\mathcal{I}_g$ becomes elevated while reaching diagnostic T1D (to the right of the black line and with $\kappa \leq 10^{-9}$), $\mathcal{I}_g$ remains elevated until disease onset (to the right of the black

line with $\kappa \in [10^{-9}, 8 \times 10^{-8}]$), $\mathcal{I}_g$ remains undetectable throughout disease progression (the blue regimes close to the right edge of each panel). The high κ value in the latter case causes β-cell destruction to be too fast for autoantibody accumulation. Most of these outcomes are consistent with experimental observations in humans and animal models that screened positive for T1D-specific autoantibodies [14, 15].

To determine how fast the level of autoantibodies rise with respect to time while varying k and κ, we quantify in panels (B1-B3) the duration (in days) for the autoantibodies to reach the following detectability levels: 0.15 (C1), 0.55 (C2) and 0.95 (C3). As demonstrated by these panels and the color bar on the right, the rise in the level of autoantibodies in most cases is very fast and reaches its maximal level of 0.95 in less than 200 days after the engagement of T-cells in the destruction of β-cells. It should be mentioned here that the blue regime in panels (C2) and (C3) for large κ, indicates that the maximal detectability levels chosen (0.55 and 0.95, respectively) are never attained in these two cases. This result could be used as a criterion to determine when high risk subjects could be tested for autoantibodies as a diagnostic tool.

The examination of the levels of β-cells (β) and autoantibodies ($\mathcal{I}_g$) in Fig. S1.2 can be better understood by tracking the time evolution of these two variables as well as T-cells ($\mathcal{T}$) in response to variations to the same key parameters k and κ. As shown in Fig. S1.3, the 30-year time evolution of $\mathcal{T}$ (A1-A4), $\mathcal{I}_g$ (B1-B4) and β (C1-C4), after the start of the autoimmune attack, are plotted as heat-maps. These heat-maps are generated by taking $k \in [10^{-4}, 1]$ and choosing the following values for the killing efficacy κ: 10^{-11} (A1-C1), 10^{-10} (A2-C2), 10^{-9} (A3-C3), 10^{-8} (A4-C4) $(\text{day}\cdot\text{cell})^{-1}$. The one consistent feature observed across all these panels is the absence of any autoimmune attack at all time when k is close to 1 (i.e., T-cell avidity is too small to invoke an autoimmune response or even illicit T-cell expansion). However, when k is large enough, T-cell expansion becomes prominent as demonstrated by the fast increase (within few months) in $\mathcal{T}$ and the appearance of red regimes in panels (A1-A4). The increase in $\mathcal{T}$ does not always imply a significant loss in β-cells. In fact, panel (C1) shows that even after 30-years of follow up, we do not see much loss in β-cells, because κ is too small for T-cells to cause any harm. By increasing κ, β-cell loss becomes significant as demonstrated by the appearance of blue regimes in panels (C2-C4). The bigger the value of κ, the faster the loss in β-cells and the quicker the manifestation of the disease. T-cell survival, in these cases, is not maintained due to the decline in β-cell specific peptides that are produced from apoptotic β-cells required for T-cell activation. The rise and decline in $\mathcal{T}$ create these (red) "waves" that are induced by the transient bistability of the model. This explains the appearance of blue regimes in panels (A3-A4) in later years which are compatible with the blue regimes in panels (C3-C4). As for the level of circulating autoantibodies, panels (B1-B4) show that the time evolution of $\mathcal{I}_g$ is similar to that of $\mathcal{T}$, except for the delay in the rise of $\mathcal{I}_g$ to its peak when compared to that of $\mathcal{T}$. This delay suggests that a major damage to β-cells could occur in susceptible individuals before they test positive for islet-specific autoantibodies.

One could interpret panels (A1-C1) and (A2-C2) to correspond to high risk subjects that test positive to autoantibodies their entire lives but never develop the disease, panels (A3-C3) to correspond to high risk subjects that become type 1 diabetic and test positive to autoantibodies almost their entire life, and panels (A4-C4) to correspond to individuals that develop the disease very

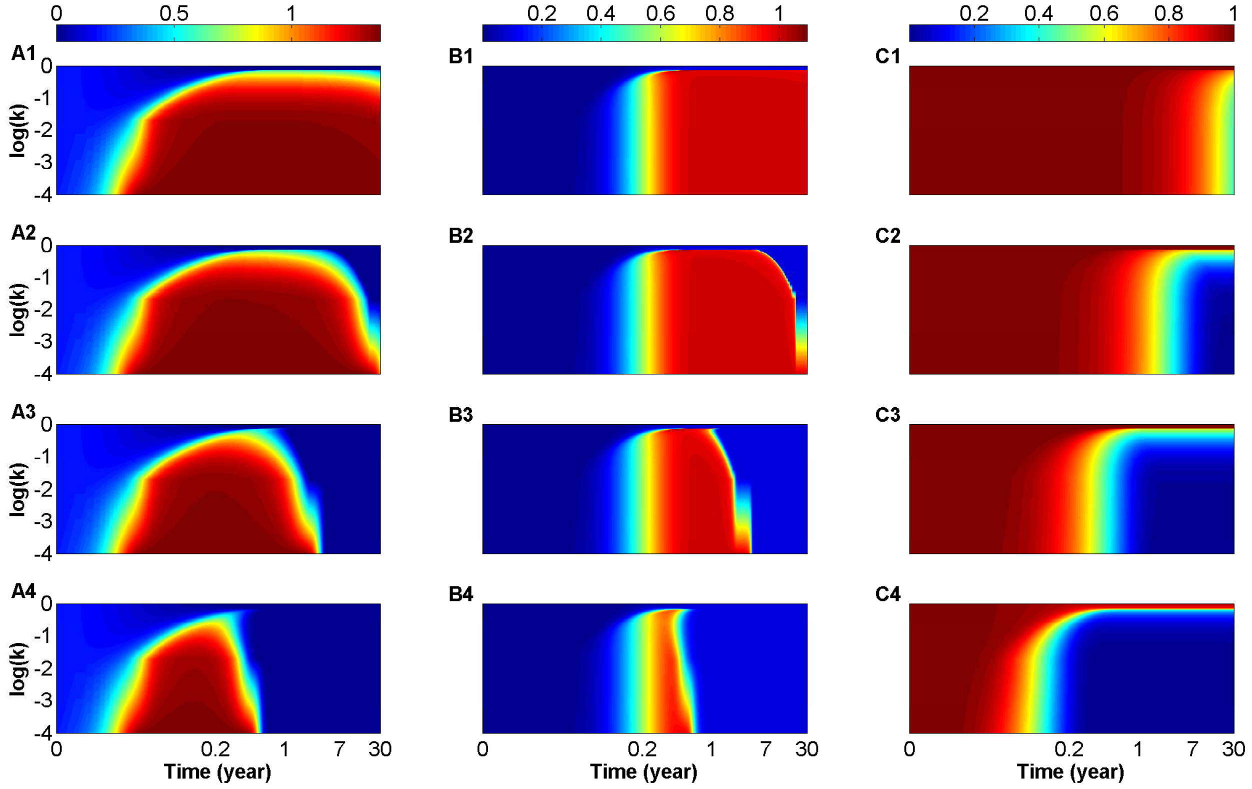

FIGURE S1.3. Time evolution of T1D disease progression in response to variations in T-cell avidity, within the range $[10^{-4}, 1]$, at various values of T-cell killing efficacy: $\kappa = 10^{-11}$ (A1-C1), $= 10^{-10}$ (A2-C2), $= 10^{-9}$ (A3-C3) and $= 10^{-8}$ (A4-C4) $(\text{day}\cdot\text{cell})^{-1}$. The levels of autoreactive T-cells $\mathcal{T}$ (A1-A4), autoantibodies $\mathcal{I}_g$ (B1-B4) and surviving β-cells (β) (C1-C4) are plotted as heat-maps with respect to both T-cell avidity k^{-1} and time over 30 years. The color-coding in each panel is quantified by the color-bars on top of each column. Notice here that increasing κ gradually increases β-cell loss and decreases the time duration of T-cell (survival) waves induced by the quasi-stability (transience) of the autoimmune state.

quickly (due to the presence of very potent and destructive T-cells), but test positive for autoantibodies during only a short window of time. Such criterion can be used to determine the risk associated with each clone of autoantibodies and the timing of T1D disease onset in individuals.

S1.2. TWO-CLONE MODEL

S1.2.1. **Model Rescaling.** We extend the one-clone model in Eqns. (S1.6)–(S1.11) by increasing the number of clones of T-cells under consideration. However, to limit the complexity of the model, we consider the simplest scenario possible in which we include two clones of T-cells with distinct autoantigenic specificities and different levels of avidity. Each one of these two clones is further divided into high and low avidity subclones. In other words, we assume that the model consists of two subclones T_{11} and T_{12} that are reactive to one autoantigen (labeled P_1) and two other subclones that are reactive to another autoantigen (labeled P_2), where $k_{22} \le k_{21} \le k_{12} \le k_{11}$.

According to this formalism, B-cells, plasma-cells and autoantibodies are also divided into two clones or copies (B_1, B_2), (P_{c_1}, P_{c_2}) and (I_{g_1}, I_{g_2}), respectively. By ignoring thymus input and setting $s = \mu = \delta_\beta = 0$, the equations describing this model are therefore given by

$$\frac{dT_{ji}}{dt} = \alpha_{ji} T_{ji} \frac{P_j}{P_j + k_{ji}} - \epsilon T_{ji}(T_{11} + T_{12} + T_{21} + T_{22}) - \delta_{T_{ji}} T_{ji} \tag{S1.28}$$

$$\frac{dB_j}{dt} = \gamma_j + \left[\left(-\eta_{2j} \mathcal{H}(T_{11}, T_{12}, T_{21}, T_{22}) + \eta_{1j}\right) \left(\frac{P_j}{P_j + k_{j1}} + \frac{P_j}{P_j + k_{j2}} \right) - \delta_{B_j} \right] B_j \tag{S1.29}$$

$$\frac{dP_{c_j}}{dt} = \eta_{2j} \mathcal{H}(T_{11}, T_{12}, T_{21}, T_{22}) \left(\frac{P_j}{P_j + k_{j1}} + \frac{P_j}{P_j + k_{j2}} \right) B_j - \delta_{P_{c_j}} P_{c_j} \tag{S1.30}$$

$$\frac{dI_{g_j}}{dt} = a_{1j} B_j + a_{2j} P_{c_j} - \delta_{I_{g_j}} I_{g_j} \tag{S1.31}$$

$$\frac{d\beta}{dt} = -\kappa \mathcal{H}(T_{11}, T_{12}, T_{21}, T_{22}) \tag{S1.32}$$

$$\frac{dP_j}{dt} = R_j \kappa \mathcal{H}(T_{11}, T_{12}, T_{21}, T_{22}) \beta - \delta_{P_j} P_j, \tag{S1.33}$$

where $j, i = 1,2$, and

$$\mathcal{H}(T_{11}, T_{12}, T_{21}, T_{22}) = r(T_{11} + \phi_1 T_{12}) + (T_{21} + \phi_2 T_{22})$$

($r < 1$ and $\phi_1, \phi_2 > 1$). Notice here that this model assumes the presence of not only intra-clonal competition between T-cells reactive to the same autoantigen, but also cross-clonal competition between T-cells that are reactive to two different autoantigens. The limited physical space available for these T-cells to bind and expand makes this assumption valid.

We simplify the model (S1.28)–(S1.33) by making the following substitutions

$$\left.\begin{array}{lllllllll} \mathcal{T}_{ji} & = & \dfrac{T_{ji}}{\hat{R}}, & \mathcal{B}_j & = & \dfrac{\delta_{B_j} B}{\gamma_j}, & \mathcal{P}_{c_j} & = & \dfrac{\delta_{P_{c_j}} P_{c_j}}{\gamma_j} \\ \mathcal{I}_{g_j} & = & \dfrac{\delta_{I_{g_j}} \delta_{P_{c_j}} I_{g_j}}{a_{2j} \gamma_j}, & \upbeta & = & \dfrac{\beta}{\beta_0}, & \mathcal{P}_j & = & \dfrac{\delta_{P_j} P_j}{R_j \kappa \hat{R} \beta_0} \end{array}\right\}, \tag{S1.34}$$

Parameter	Description	Default Value
$\alpha_{11},\alpha_{12},\alpha_{21},\alpha_{22}$	Expansion rates of $\mathcal{T}_{11},\mathcal{T}_{12},\mathcal{T}_{21},\mathcal{T}_{22}$, resp.	$10,6,4,2$ day^{-1}
$\delta_{T_{11}},\delta_{T_{12}},\delta_{T_{21}},\delta_{T_{22}}$	Turnover rates of $\mathcal{T}_{11},\mathcal{T}_{12},\mathcal{T}_{21},\mathcal{T}_{22}$, resp.	$0.1,0.15,0.1,0.2$ day^{-1}
$\mathrm{k}_{11},\mathrm{k}_{12},\mathrm{k}_{21},\mathrm{k}_{22}$†	pMHC-expression level for 50% maximum activation levels of $\mathcal{T}_{11},\mathcal{T}_{12},\mathcal{T}_{21},\mathcal{T}_{22}$, resp.	$2,1.5,0.26,0.1$
$\delta_{B_1},\delta_{B_2}$	Turnover rates of $\mathcal{B}_1,\mathcal{B}_2$, resp.	$0.02,0.04$ day^{-1}
η_{11},η_{12}	Expansion rates of $\mathcal{B}_1,\mathcal{B}_2$, resp.	$9.1,4.55\times10^{-3}$ day^{-1}
$\mathrm{\eta}_{21},\mathrm{\eta}_{22}$	Maturation rates of $\mathcal{B}_1,\mathcal{B}_2$, resp.	$7.36,8.28$ day^{-1}
$\delta_{P_{c_1}},\delta_{P_{c_2}}$	Turnover rates of $\mathcal{P}_{c_1},\mathcal{P}_{c_2}$, resp.	$0.2,0.4$ day^{-1}
$\delta_{I_{g_1}},\delta_{I_{g_1}}$	Degradation rates of $\mathcal{I}_{g_1},\mathcal{I}_{g_2}$, resp.	$0.001,0.034$ day^{-1}
$\mathrm{a}_1,\mathrm{a}_2$	B-to-plasma autoantibody-release ratios for $\mathcal{I}_{g_1},\mathcal{I}_{g_2}$, resp.	$0.1,0.1$
$\delta_{P_1},\delta_{P_2}$	Degradation rates of $\mathcal{P}_1,\mathcal{P}_2$, resp.	$0.1,0.1$ day^{-1}
r	Relative effects of $\mathcal{T}_{1i}$-to-$\mathcal{T}_{2i}$, $(i=1,2)$	0.7
ϕ_1,ϕ_2	Relative effects of $\mathcal{T}_{11}$-to-$\mathcal{T}_{12}$ and $\mathcal{T}_{21}$-to-$\mathcal{T}_{22}$, resp.	$1.25,3$

TABLE S1.2. Parameter values of the (scaled) two-clone model (S1.35)–(S1.40). †The ranges of pMHC-expression level are listed in (3) in the main text.

where $\hat{R}=(\alpha_{21}^{1/2}-\delta_{21}^{1/2})^2/\epsilon$. The model then becomes

$$\frac{d\mathcal{T}_{ji}}{dt}=\alpha_{ji}\mathcal{T}_{ji}\frac{\mathcal{P}_j}{\mathcal{P}_j+\mathrm{k}_{ji}}-(\alpha_{21}^{1/2}-\delta_{21}^{1/2})^2\mathcal{T}_{ji}(\mathcal{T}_{11}+\mathcal{T}_{12}+\mathcal{T}_{21}+\mathcal{T}_{22})-\delta_{T_{ji}}\mathcal{T}_{ji} \tag{S1.35}$$

$$\frac{d\mathcal{B}_j}{dt}=\delta_{Bj}+\left[\left(-\mathrm{\eta}_{2j}\mathcal{H}(\mathcal{T}_{11},\mathcal{T}_{12},\mathcal{T}_{21},\mathcal{T}_{22})+\eta_{1j}\right)\left(\frac{\mathcal{P}_j}{\mathcal{P}_j+\mathrm{k}_{j1}}+\frac{\mathcal{P}_j}{\mathcal{P}_j+\mathrm{k}_{j2}}\right)-\delta_{B_j}\right]\mathcal{B}_j \tag{S1.36}$$

$$\frac{d\mathcal{P}_{c_j}}{dt}=\delta_{P_{cj}}\left[\frac{\mathrm{\eta}_{2j}}{\delta_{B_j}}\mathcal{H}(\mathcal{T}_{11},\mathcal{T}_{12},\mathcal{T}_{21},\mathcal{T}_{22})\left(\frac{\mathcal{P}_j}{\mathcal{P}_j+\mathrm{k}_{j1}}+\frac{\mathcal{P}_j}{\mathcal{P}_j+\mathrm{k}_{j2}}\right)\mathcal{B}_j-\mathcal{P}_{c_j}\right] \tag{S1.37}$$

$$\frac{d\mathcal{I}_{g_j}}{dt}=\delta_{I_{gj}}\left[\mathrm{a}_j\mathcal{B}_j+\mathcal{P}_{c_j}-\mathcal{I}_{g_j}\right] \tag{S1.38}$$

$$\frac{d\mathrm{\beta}}{dt}=-\kappa\hat{R}\mathcal{H}(\mathcal{T}_{11},\mathcal{T}_{12},\mathcal{T}_{21},\mathcal{T}_{22})\mathrm{\beta} \tag{S1.39}$$

$$\frac{dP_j}{dt}=\delta_{P_j}\left[\mathcal{H}(\mathcal{T}_{11},\mathcal{T}_{12},\mathcal{T}_{21},\mathcal{T}_{22})\mathrm{\beta}-\mathcal{P}_j\right], \tag{S1.40}$$

where the new non-italicized parametric quantities introduced into Eqns. (S1.35)–(S1.40) satisfy

$$\mathrm{k}_{ji}=\frac{\delta_{P_j}k_{ji}}{R_j\kappa\hat{R}\beta_0},\quad \mathrm{\eta}_{2j}=\eta_{2j}\hat{R},\quad \mathrm{a}_j=\frac{a_{1j}\delta_{P_{cj}}}{a_{2j}\delta_{B_j}}.$$

Once again, we assume here that $\mathrm{k}_{22}\le\mathrm{k}_{21}\le\mathrm{k}_{12}\le\mathrm{k}_{11}$. The simulations presented in this paper are all generated using these equations and parameter values listed in Table S1.2.

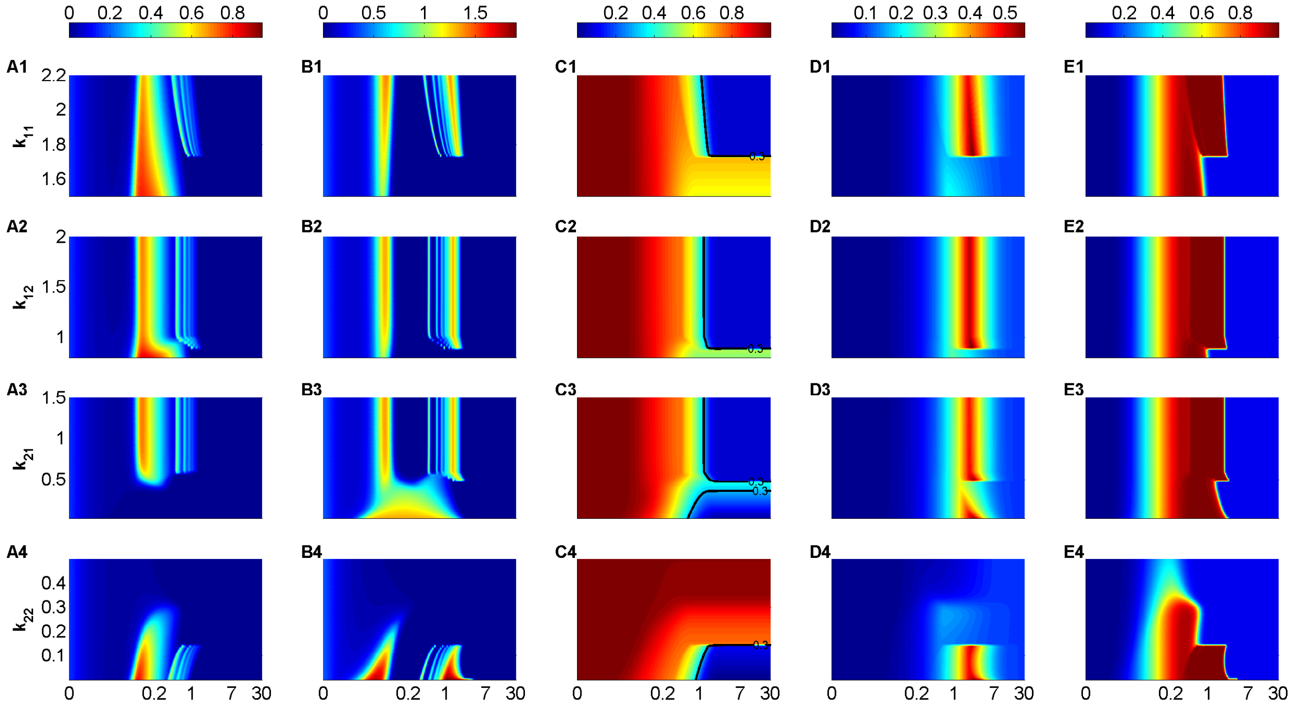

FIGURE S1.4. Heat-map simulations of the (scaled) two-clone model (S1.35)–(S1.40) for a high value of T-cell killing efficacy $\kappa = 10^{-8}$ (day·cell)$^{-1}$. The 30-year time evolution of $r(\mathcal{T}_{11} + \phi_1 \mathcal{T}_{12})$ (A1-A4), $\mathcal{T}_{21} + \phi_2 \mathcal{T}_{22}$ (B1-B4), β (C1-C4), $\mathcal{I}_{g_1}$ (D1-D4) and $\mathcal{I}_{g_2}$ (E1-E4). The colors in these panels represent the levels of these quantities according to the color-bars on top of each column, whereas the black lines in panels (C1-C4) represent the 0.3-critical threshold. The simulations are performed over the following ranges of T-cell avidities: $k_{11} \in [1.4, 2.2]$ (A1-E1), $k_{12} \in [0.8, 2]$ (A2-E2) $k_{21} \in [0.026, 1.5]$ (A3-E3) and $k_{22} \in [10^{-4}, 0.8]$ (A4-E4), where the inequality $k_{11} \geq k_{21} \geq k_{12} \geq k_{22}$ is always satisfied. As shown, the oscillations in T-cell level persist at larger values of κ and the loss of β-cells is very fast and substantial.

S1.2.2. **Model Simulations.** The heat-map simulations of the (scaled) two-clone model displayed in Fig. S1.4 show how varying k_{ji}, $j, i = 1, 2$, within their perspective ranges specified in the caption, affect the 30-year time evolution of the model. By taking $\kappa = 10^{-8}$ (day·cell)$^{-1}$, we find that the scaled level of the effective population sizes of $\mathcal{P}_1$-specific T-cells: $r(\mathcal{T}_{11} + \phi_1 \mathcal{T}_{12})$ (A1-A4) and $\mathcal{P}_2$-specific T-cells: $\mathcal{T}_{21} + \phi_2 \mathcal{T}_{22}$ (B1-B4) (both of which are measures of avidity maturation), produce cyclic fluctuations and successive waves for certain k_{ji} values. Since T-cell killing efficacy is assumed high here, the decline in the scaled level of β-cells β (C1-C4) is very prominent and fast, crossing the 0.3-critical threshold, shown as black lines, in less than 1 year. Furthermore, panels (E1-E4) show that the scaled level of $\mathcal{P}_2$-specific autoantibodies appear before $\mathcal{P}_1$-specific autoantibodies (D1-D4) and survive longer in circulation.

Figure S1.4 also demonstrates that it is possible to improve disease outcomes (i.e., keep β above 0.3) by increasing the avidity of either $\mathcal{T}_{11}$ (C1) or $\mathcal{T}_{12}$ (C2), or by decreasing the avidity of $\mathcal{T}_{22}$

(C4). The narrow regime with elevated level of β between the two black lines in (C3), representing the 0.3-critical threshold, is invoked by solution trajectories propagating close to the stable manifold of the transient saddle point. Although it is possible theoretically to target this regime to improve disease outcomes, its sensitivity to perturbations makes this clinically difficult, especially in later years when the regime is narrower.

REFERENCES

[1] Skowera A, Ellis RJ, Varela-Calviño R, Arif S, Huang GC, et al. (2008) CTLs are targeted to kill β cells in patients with type 1 diabetes through recognition of a glucose-regulated preproinsulin epitope. J Clin Invest 118: 3390-3402.

[2] Standifer NE, Ouyang Q, Panagiotopoulos C, Verchere CB, Tan R, et al. (2006) Identification of Novel HLA-A*0201-restricted epitopes in recent-onset type 1 diabetic subjects and antibody-positive relatives. Diabetes 55: 3061-3067

[3] Khadra A, Pietropaolo M, Nepom GT, Sherman A (2011) Investigating the role of T-cell avidity and killing efficacy in relation to type 1 diabetes prediction. PLoS ONE 6(5): e14796.

[4] Khadra A, Santamaria P, Edelstein-Keshet L (2009) The role of low avidity T cells in the protection against type 1 diabetes: A modeling investigation. J Theor Biol 256: 126-141.

[5] Mahaffy JM, Edelstein-Keshet L (2007) Modeling cyclic waves of circulating T cells in autoimmune diabetes. SIAM J Applied Math (SIAP) 67: 915-1937.

[6] Khadra A, Santamaria P, Edelstein-Keshet L (2010) The pathogenicity of self-antigen decreases at high levels of autoantigenicity: a computational approach. Int Immunol 22: 571-582.

[7] Khadra A, Tsai S, Santamaria P, Edelstein-Keshet L (2010) On how monospecific memory-like autoregulatory CD8$^+$T cells can blunt diabetogenic autoimmunity: A computational approach. J Immunol 185: 5962-5972.

[8] Kim P, Lee P, Levy D (2007) Modeling regulation mechanisms in the immune system. J Theor Biol 246: 33-69.

[9] Standifer NE, Burwell EA, Gersuk VH, Greenbaum CJ, Nepom GT (2009) Changes in autoreactive T cell avidity during type 1 diabetes development. Clin Immunol 132: 312-320.

[10] Vernino L, McAnally LM, Ramberg J, Lipsky PE (1992) Generation of nondividing high rate Ig-secreting plasma cells in cultures of human B cells stimulated with anti-CD3-activated T cells. J Immunol 148: 404-410.

[11] Parham P (2009) The immune system. Garland Science, 3d edition. 608p.

[12] Edelstein-Keshet L (2005) Mathematical models in biology (Classics in Applied Mathematics). Classics in Applied Mathematics (Book 46). New York, Random House. SIAM: Society for Industrial and Applied Mathematics; 1 edition.

[13] Strogatz SH (2001) Nonlinear dynamics and chaos: With applications to Physics, Biology, Chemistry, and Engineering (studies in nonlinearity). Cambridge, MA, Westview Press; 1 edition.

[14] Morran MP, Casu A, Arena VC, Pietropaolo S, Zhang YJ, et al. (2010) Humoral autoimmunity against the extracellular domain of the neuroendocrine autoantigen IA-2 heightens the risk of type 1 diabetes. Endocrinology 151: 2528-2537.

[15] Miao D, Yu L, Eisenbarth GS (2007) Role of autoantibodies in type 1 diabetes. Front Biosci 12: 1889-1898.
